# The impact of caring for dying patients in intensive care units on a physician’s personhood: a systematic scoping review

**DOI:** 10.1186/s13010-020-00096-1

**Published:** 2020-11-25

**Authors:** Joshua Tze Yin Kuek, Lisa Xin Ling Ngiam, Nur Haidah Ahmad Kamal, Jeng Long Chia, Natalie Pei Xin Chan, Ahmad Bin Hanifah Marican Abdurrahman, Chong Yao Ho, Lorraine Hui En Tan, Jun Leng Goh, Michelle Shi Qing Khoo, Yun Ting Ong, Min Chiam, Annelissa Mien Chew Chin, Stephen Mason, Lalit Kumar Radha Krishna

**Affiliations:** 1 Yong Loo Lin School of Medicine, National University of Singapore, Singapore, Singapore; 2 Division of Supportive and Palliative Care, National Cancer Centre Singapore, Singapore, Singapore; 3 Division of Cancer Education, National Cancer Centre Singapore, Singapore, Singapore; 4 Medical Library, National University of Singapore Libraries, National University of Singapore, Singapore, Singapore; 5 Palliative Care Institute Liverpool, Academic Palliative & End of Life Care Centre, Cancer Research Centre, University of Liverpool, 200 London Road, Liverpool, L3 9TA UK; 6 Duke-NUS Graduate Medical School, Singapore, Singapore; 7 Centre for Biomedical Ethics, National University of Singapore, Singapore, Singapore; 8 PalC, The Palliative Care Centre for Excellence in Research and Education, Singapore, Singapore

**Keywords:** Intensive care unit (ICU), Physicians, Death and dying, Ring theory of personhood (RToP), Personhood, Resilience

## Abstract

**Background:**

Supporting physicians in Intensive Care Units (ICU)s as they face dying patients at unprecedented levels due to the COVID-19 pandemic is critical. Amidst a dearth of such data and guided by evidence that nurses in ICUs experience personal, professional and existential issues in similar conditions, a systematic scoping review (SSR) is proposed to evaluate prevailing accounts of physicians facing dying patients in ICUs through the lens of Personhood. Such data would enhance understanding and guide the provision of better support for ICU physicians.

**Methods:**

An SSR adopts the Systematic Evidenced Based Approach (SEBA) to map prevailing accounts of caring for dying patients in ICUs. To enhance the transparency and reproducibility of this process, concurrent and independent use of tabulated summaries, thematic analysis and directed content analysis (Split Approach) is adopted.

**Results:**

Eight thousand three hundred fifty-eight abstracts were reviewed from four databases, 474 full-text articles were evaluated, 58 articles were included, and the Split Approach revealed six categories/themes centered around the Innate, Individual, Relational and Societal Rings of Personhood, conflicts in providing end of life care and coping mechanisms employed.

**Conclusion:**

This SSR suggests that caring for dying patients in ICU impacts how physicians view their personhood. To resolve conflicts within individual concepts of personhood, physicians use prioritization, reframing and rely on accessible, personalized support from colleagues to steer coping strategies. An adapted form of the Ring Theory of Personhood is proposed to direct timely personalized, appropriate and holistic support.

**Supplementary information:**

The online version contains supplementary material available at 10.1186/s13010-020-00096-1.

## Background

The COVID-19 pandemic has overwhelmed healthcare systems and has physicians facing unprecedented levels of death and dying in Intensive Care Units (ICU)s [1, 2]. Received knowledge suggests that these experiences impact the emotional, psychological and physical wellbeing of ICU physicians, though there is little understanding of how these complex, frequently and closely related issues arise, much less are supported [3–5]. This dearth of data has raised concerns about how ICU physicians cope amidst the COVID-19 pandemic.

In the face of data suggesting that nurses in ICU report similar personal, professional and existential issues in comparable circumstances [6–12], a systematic scoping review (SSR) was proposed to study accounts on the impact of caring for dying patients on physicians in the adult ICU in the extent literature. Better understanding of how ICU physicians cope with death and dying could inform efforts to support them during the pandemic and beyond.

## Methods

**Fig. 1:**
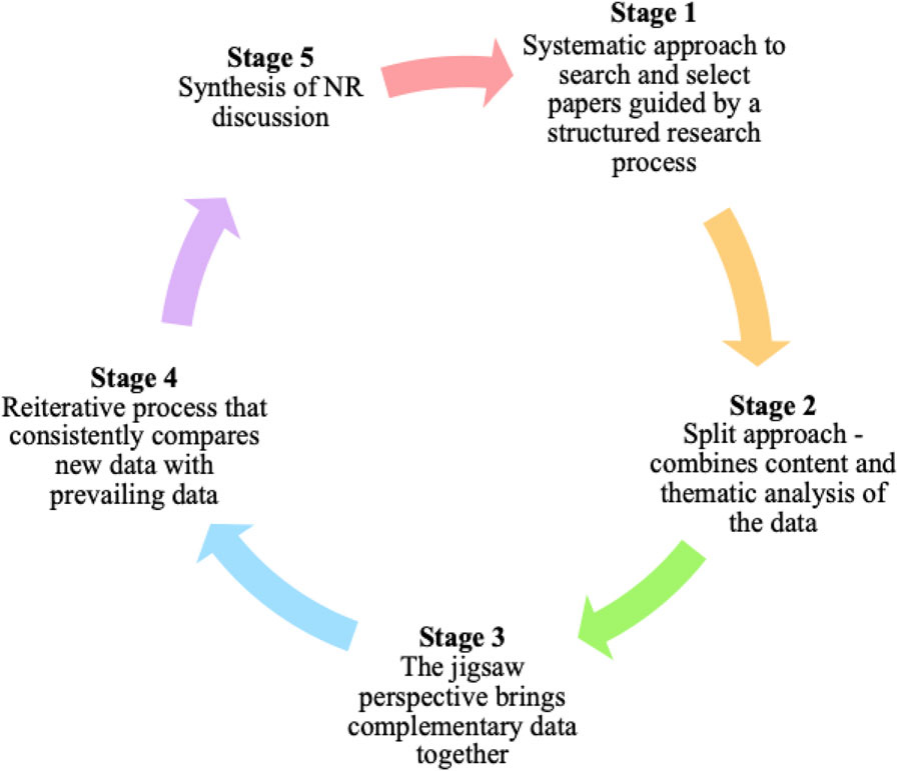
The SEBA Approach

An SSR is proposed to map current data on the subject. To enhance its reproducibility and transparency, the Systematic Evidenced Based Approach (SEBA) was used to guide the SSR (henceforth SSR in SEBA). The SSR in SEBA’s constructivist perspective captures contextual factors whilst a relativist lens facilitates the inculcation of diverse experiences and perspectives of ICU physicians caring for dying patients [13–16]. The research team was supported by medical librarians from the Yong Loo Lin School of Medicine (YLLSoM) at the National University of Singapore and National Cancer Centre Singapore (NCCS), and local experts and clinicians at NCCS, the Palliative Care Institute Liverpool, YLLSoM and Duke-NUS Medical School (henceforth the expert team) to enhance the reproducibility, transparency and trustworthiness of this analysis. The principles of interpretivist analysis were employed in the 5 stages of SEBA highlighted in Fig. 1.

### Stage 1 of SEBA: systematic approach

#### Determining the title and research question

To ensure a systematic approach, the research and expert teams established the goals of the SSR and the population, context and concept (PCC) to be evaluated. The primary research question was determined to be: “what is known of how physicians contend with death and dying in the adult ICU setting?” and the secondary questions: “what is the impact of caring for dying patients on physicians in the adult ICU?” and “how do these effects manifest?”

#### Inclusion criteria

A PICOS format was adopted to guide the research process as shown in Table 1 [17, 18].

**Table 1:** PICOS, inclusion criteria and exclusion criteria applied to literature search

	Inclusion criteria	Exclusion criteria
Population	• Physicians	• Main focus on other health professionals such as:
		° Nurses
		° Allied health workers
		° Healthcare support staff
		• Main focus on **patients**, caregiver, family or friends
		• Students from health professions such as:
		° Medical students
		° Nursing students
		° Allied health students
Intervention / Exposure	• Being involved in the care of dying patients in the adult ICU	• No involvement in care of dying patients
		° No clearly defined patient care experience (e.g. study just explores attitudes to death/ palliative care)
		° Patient population not dying patients (incl. “geriatrics”, patients without specification that they are dying)
		° Physician assisted suicide/ medical assistance in death/ suicide
		• Personal experience of death of family/ friend
		• Non-adult ICUs such as:
		° Paediatric ICUs
		° Neonatal ICUs
Comparison		
Outcome measures	• Impact on doctors ° Emotional ° Psychological ° Behavioural ° Physical	
Study design	• English language • Time of publication between 1990 and 2019 • No restriction on study design (qualitative, quantitative, mixed) • No restriction on geographical location of study or publication	• Grey Literature, electronic and print information not controlled by commercial publishing • Narrative literature reviews without methodology • Case reports and series, commentaries, editorials, and perspectives • Non-English publications without English translation • Unable to retrieve full article

#### Searching

The 11 members of the research team carried out independent searches of four bibliographic databases (PubMed, Embase, CINAHL, and PSYCINFO) for articles published between 1st January 1990 and 31st December 2019. The searches were carried out between 13th February 2020 and 24th April 2020. The PubMed search strategy may be found in Additional file 1.

Each member of the research team independently sieved through all titles and abstracts from the individual searches of the four databases and created their own lists of titles to be reviewed. Comparing these individual lists via online meetings, the teams used ‘negotiated consensual validation’ to achieve consensus on the final list of titles to be reviewed [19].

The research team then independently reviewed each of the full-text articles from this final list, created individual lists of articles to be included, discussed them online and achieved a consensus on the final list of full-text articles to be included in the SSR. The results of this process are outlined below.

## Results

**Fig. 2:**
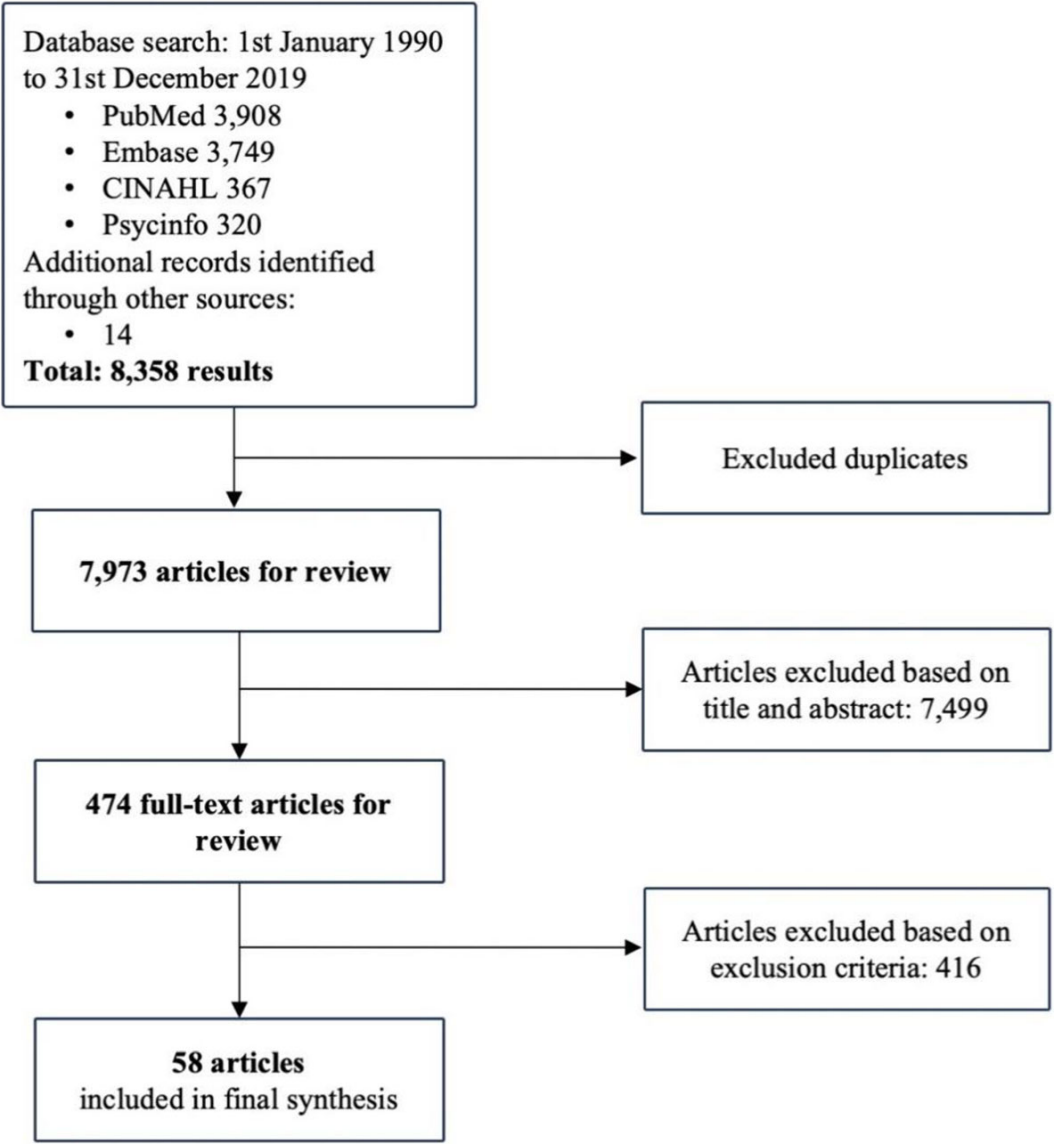
PRISMA Flow Chart

Eight thousand three hundred fifty-eight abstracts were identified from four databases, 7973 articles were reviewed, and 58 articles were included as shown in Fig. 2.

### Stage 2 of SEBA: Split approach

Three teams of at least three researchers independently reviewed the 58 included full-text articles.

The first team summarized and tabulated them as recommended by Wong, Greenhalgh [20]‘s RAMESES publication standards: meta-narrative reviews and Popay, Roberts [21]‘s “Guidance on the conduct of narrative synthesis in systematic reviews”. The tabulated summaries ensure that key points of the articles are not lost (Additional file 2). The team also evaluated the quality of quantitative and qualitative studies included in this review using the Medical Education Research Study Quality Instrument (MERSQI) [22] and the Consolidated Criteria for Reporting Qualitative Studies (COREQ) [23].

Concurrently, the second team independently analyzed the 58 articles using Braun and Clarke [24]‘s approach to thematic analysis while the third team adopted Hsieh and Shannon [25]‘s approach to directed content analysis. Enhancing the reliability of the analyses, concurrent thematic and directed content analysis is a key feature of the ‘Split Approach’.

The narrative produced by consolidating the tabulated summaries, themes and categories was guided by the Best Evidence Medical Education (BEME) Collaboration guide [26] and the STORIES (STructured apprOach to the Reporting In healthcare education of Evidence Synthesis) statement [27].

#### Themes identified using thematic analysis

The themes identified were the impact of caring for the dying on one’s self; relationships; interactions; conflicts in providing end of life (EoL) care and the coping strategies employed. Elements of each of these themes are featured in Table 2.

**Table 2:** Summary of Thematic Analysis and Direct Content Analysis

Themes and examples	Categories and Example
**Theme 1: Self**	**Category 1: Innate Ring**
**Emotion**	**Perception of life and death**
1. Internal conflict	1. Confrontation with own mortality
2. Management of own expectations	2. Conception of a good death impacting end-of-life care
3. Confrontation with own mortality	3. One has a right to die
4. Apprehension/Distress	**Category 2: Individual Ring**
5. Fear due to unintentional transference to own family members	**Ability to make sense of things**
6. Satisfaction in providing end-of-life care	1. Impact ability to make decisions
**Thoughts**	**Abilities to communicate**
1. Doubt	1. Loss of ability to communicate and relate to patients
2. Perception of emotional involvement	2. Improvement in communication skills
3. Professional responsibility	**Abilities to express feelings**
4. Death of a patient perceived to be a personal failure	1. Emotional detachment
5. Death of a patient not perceived to be a personal failure	2. Emotion connection
6. Intervention as prolonging suffering for patients	**Acquired ability**
7. Intervention as prolonging suffering for patient’s family	1. Lack of knowledge about end-of-life
8. Withdrawal of treatment as life-shortening	2. Inadequate opportunities for end-of-life care training
9. Decision between active treatment or palliative intention	3. Doubt and lack of confidence in clinical skills
10. Perception that nurses do not grasp the complexity of end-of-life decision making	4. Testing of practical skills such as treatment withdrawal techniques
11. Motivated to improve communication skills	5. Acquisition of new skills with experience
12. Perception of intensive care unit as not conducive for palliative care discussions	**Beliefs**
**Behavior**	*Personal Beliefs*
1. Impaired ability to make decisions	1. Dilemmas about the balancing of opposing values
2. Impaired ability to communicate	2. Personal beliefs reflected in end-of-life practices and communication
3. Emotional detachment	*Exposed to Ethical dilemmas such as:*
4. Difficulty and discomfort when broaching topic of death to patients	1. Differences in ethical opinion surrounding treatment withholding and withdrawal
5. Attempts to avoid discussion of death in general	2. Futile treatment
6. Fear of litigation leading to defensive practice	3. Lack of advanced directives and families’ aggressive care requests causing moral distress
7. Adherence to decisions despite potential legal kickback	*Religious views*
8. Personal, patient, institutional and societal factors affecting decision making	1. Influenced end-of-life discussion and decision making
9. Poor translation of spiritual ideas to goals of care	2. Did not influence end-of-life practices
**Theme 2: Relationships**	**Perceived role as a doctor**
**Physician’s family**	1. Perceived duty to prolong life causing moral distress
1. Fear due to unintentional transference to own family members	2. Uncertainty about role in end-of-life discussions resulting in no/late end-of-life discussion
**Theme 3: Interactions**	3. Paternalistic approach to decisionmaking
**Patients**	4. Satisfaction upon reconciling dual role of saving lives and managing death well
1. Challenges during end-of-life communication	**Category 3: Relational Ring**
2. Managing expectations of patients	**Family**
3. Inspiring interactions with patients	1. Fear due to unintentional transference to own family members
**Patient’s Family**	**Category 4: Societal Ring**
1. Experiencing conflict with patient’s family	**Physical environment**
2. Effects of conflict on the relationship	1. Availability of resources in different countries influencing end-of-life care
3. Family’s concern for patient’s possible pain and distress	**Cultural environment**
4. Managing expectations of patient’s family	1. Physician’s end-of-life care attitudes, behaviors and decisions privy to cultural norms
5. Family’s distress after end-of-life care discussion	2. Death and dying perceived as a “taboo” topic in certain cultures
6. Empowering interactions with patient’s family	3. Need for end-of-life care to be sensitive to different cultures encountered
7. Factors affecting communication	4. Workplace culture impacting attitudes and practices
8. Creation of soft landing when informing patient’s family about death	**Societal expectations**
9. Perception of intensive care unit as not conducive for palliative care discussions	1. Societal expectations promoting survival and death prevention leading to negative perception of treatment withdrawal as the taking of a patient’s life, affecting physician’s end-of-life decision making
**Nurses & ICU Team**	**Legal standards**
1. Conflict between physician and intensive care unit nurses	1. Fear of litigation leading to defensive practice
2. Perception that nurses do not grasp the complexity of end-of-life decision making	2. Adherence to decisions despite potential legal kickback
3. Receiving support from other intensive care unit physicians in managing end-of-life decisions	3. Unclear laws surrounding end-of-life practices breeding legal uncertainty
**Physicians from other specialties**	**Professional Relationships**
1. Challenges with interactions	1. Conflict relating to end-of-life decisions with patient’s family and other healthcare professionals
2. Lack of understanding of one another’s role	2. Positive professional relationships
**Theme 4: Conflicts in providing end-of-life care**	**Professional standards**
**Societal Culture**	1. Professional expectation that doctors should not cause death or harm to patients
1. Societal culture impacting decision making	2. Responsibility of treatment withdrawal decision going against physician’s perceived professional standards
2. Stigma associated with death or talking about death	
**Workplace Culture**	
1. Shapes the way doctors view death	
**ICU Environment**	
1. Suitability for palliative care teaching	
2. Intensive care unit as an inappropriate place to die	
**Legal environment**	
1. Uncertainty with regards to legal implication of end-of-life practice	
**Theme 5: Coping strategies**	
**Personal strategies**	
1. Effective communication to strengthen decision making position	
2. Gaining confidence through experience and with end-of-life discussions	
3. Taking breaks from the intensive care unit or practicing on other sites	
**Strategies with patients**	
1. Collaboration with patients to reduce moral burden of decision making	
**Strategies with patient’s family**	
1. Creation of soft landing when informing patient’s family about death	
2. Collaboration with patient’s family to reduce moral burden of decision making	
**Strategies with colleagues**	
1. Conflict management interventions	
2. Emotional and experiential sharing of caring for dying patients	
3. Collaboration with interdisciplinary team members	

#### Categories identified using directed content analysis

Positing that ICU physicians will be as similarly affected as their nursing colleagues [6–12] and will likely face changes in the manner that they view themselves, their relationships with family, friends, patients, and their changing roles [6–12], the research and expert teams adopted Radha Krishna and Alsuwaigh [28]‘s Ring Theory of Personhood (RToP), an evidence-based framework to studying concepts of personhood – or “what makes you, you” to study the effects of caring for dying patients in ICU on physicians.

**Fig. 3:**
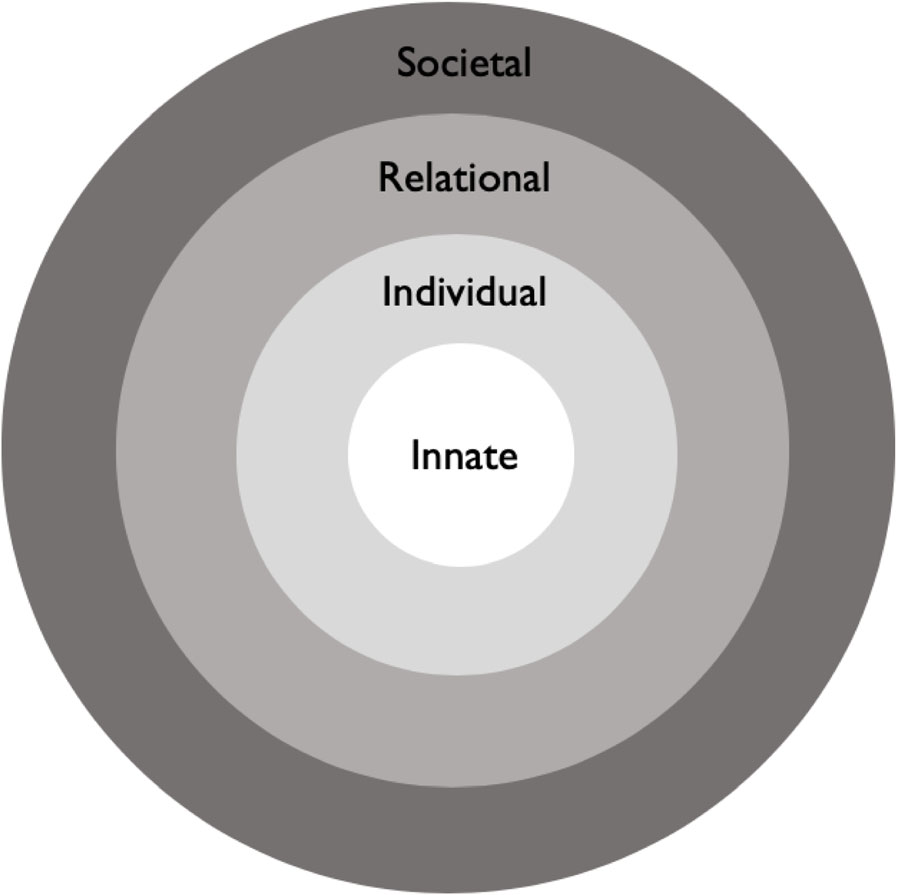
Krishna and Alsuwaigh (2015)‘s Ring Theory of Personhood

The RToP consists of four aspects of personhood which form a complex interplay with one another as shown in Fig. 3.

At the core of the Ring Theory is the Innate Ring. Krishna and Alsuwaigh (2015) defined the Innate Ring as being present for all humans by virtue of their Divine connections and/or their genetic propensity to being a human. This Innate Ring serves a critical role in ensuring that the individual is treated with the dignity and care afforded all human beings until death.

The Individual Ring comprises the particular characteristics of the individual, such as their values, beliefs, and personality, as well as their conscious functions such as their abilities, thoughts, emotions and actions.

The Relational Ring consists of important personal relationships to the individual, such as ones with loved ones and close friends. These ties are determined by the person themselves and can change over time.

The Societal Ring is the outermost ring that consists of less intimate relationships as well as societal, religious, professional and legal expectations set out in the individual’s society to guide and police conduct.

Adopting the RToP given its use in the end of life setting, the research team drew codes and categories from its description. Deductive category application [29] revealed four categories corresponding to the impact on the Innate, Individual, Relational and Societal Rings of Personhood Further elaboration is offered in Table 2 (without references) and in Additional file 3 (with references).

### Stage 3 of SEBA: funneling

A funneling approach was adopted to streamline results from the three aspects of the Split Approach. It sees data compared and combined to reduce overlap and repetition whilst retaining a holistic perspective of the data. Critical aspects are identified and a jigsaw perspective is applied to put together ‘complementary pieces’ to generate a comprehensive map of the prevailing data on the impact of caring for dying patients on ICU physicians.

### Stage 4 of SEBA: the reiterative process

Themes/categories identified were discussed with the expert team and with other local educationalists and clinicians. This process saw the research and the expert teams identify three themes (the impact of caring for dying patients on one’s self, relationships and interactions) to be similar to categories identified from the content analysis (the Innate, Individual, Relational and Societal Rings). However, as strands of the theme labeled ‘interactions’ involve aspects that move beyond the Societal Ring, an additional theme labeled ‘conflicts in providing EoL (end of life) care’ was created. As such, this saw the combined results and presentation of six themes/categories which correspond to the four rings of RToP, conflicts in providing EoL care and coping strategies.

#### Comparisons between tabulated summaries, thematic analysis and directed content analysis

Combined themes after the funneling process are presented in Table 3 (without references) and in Additional file 4 (with references). Given that many of the ‘codes’ within these category/ themes are poorly described, they have been tabulated to help analysis. To clarify, Category/ Theme 1 to 4 discuss general accounts of caring for dying patients in ICU whilst Category/ Theme 5 relates to specific accounts of experiences providing EoL care in ICU. Category/ Theme 6 review accounts of coping strategies adopted by ICU physicians.

**Table 3:** Combined Categories/ Themes

Subcategory	Elaborations
**Category/ Theme 1: Innate Ring**	
1. Perception of life and death	a) Confrontation with own mortality
	b) Conception of a good death impacting end-of-life care
	c) Death of patient perceived to be a personal failure
	d) Death of patient not perceived to be a personal failure
	e) Conflict about prolonging life as it prolonged suffering
	f) One has a right to die
**Category/ Theme 2: Individual Ring**	
1. Ability to make sense of things	*Impaired end-of-life decision making:*
	a) Personal factors
	b) Patient factors
	c) Institutional factors
	d) Societal culture
	*Doubt:*
	e) Doubt in end-of-life decision making
	f) Doubt about assessment of patient’s prognosis
	g) Doubt due to uncertainties in patient’s trajectories
	h) Internal conflict when balancing care goals
	i) Internal conflict when managing own expectations
	j) Dilemmas about active treatment versus palliative intention
2. Ability to communicate and relate	a) Loss of ability to communicate and relate to patients
	b) Poor communication skills
	c) Difficulty and discomfort when broaching topic of death to patients
	d) Attempts to avoid discussion of death in general
	e) Improvement in communication skills
	f) Confidence in ability to navigate difficult conversations
	g) Motivated to further improve communication skills
3. Ability to express feelings	a) Emotional detachment
	b) Emotions perceived as hinderance to job
	*Apprehension/Distress:*
	c) From end-of-life care
	d) From communication with family
	e) From belief that futile treatment prolonged dying process
	f) From possibility of litigation
	g) Fear due to unintentional transference to own family
	h) Emotional involvement being considered as valuable
	i) Satisfaction in involvement in patient’s end-of-life care
4. Acquired ability	a) Lacking knowledge about end-of-life
	b) Inadequate opportunities for end-of-life care training
	c) Doubt and lack of confidence in clinical skills
	d) Testing of practical skills such as treatment withdrawal techniques
	e) End-of-life decision making differing with years of experience
	f) Acquisition of new skills with experience
	g) Adequate end-of-life care training
5. Beliefs	*Personal Beliefs*
	a) Conflicting beliefs resulting in distress
	b) Dilemmas about the balancing of opposing values
	c) Personal beliefs reflected in end-of-life practices and communication
	*Ethical dilemmas*
	d) Differences in ethical opinion surrounding treatment withholding and withdrawal
	e) Futile treatment
	f) Lack of advanced directives and families’ aggressive care requests causing moral distress
	*Religious views*
	g) Influenced end-of-life discussion and decision making
	h) Did not influence end-of-life practices
6. Perceived Role as a doctor	a) To care for dying patients
	b) Perceived duty to prolong life causing moral distress
	c) Death of patient perceived to be a professional failure
	d) Death of patient not perceived to be a professional failure
	e) Uncertainty about role in end-of-life discussions resulting in no/late end-of-life discussion
	f) Paternalistic approach to decision making
	g) Perceived professional duty to collaborate and care for needs of patient’s family
	h) Professional satisfaction from caring for dying patients
	i) Satisfaction upon reconciling dual role of saving lives and managing death well
	j) Emotions perceived as hinderance to role as doctor
**Category/ Theme 3: Relational Ring**	
1. Family	a) Fear due to unintentional transference to physician’s own family members
**Category/ Theme 4: Societal Ring**	
1. Physical environment	a) Availability of resources in different countries influencing end-of-life care
	b) Intensive care unit environment as not conducive for end-of-life discussions
	*Intensive care unit as an inappropriate place to die*
	c) Lack of privacy
	d) Focus of care not allowing for palliative care
	*Suitability for palliative care teaching*
	e) Not suitable
	f) Suitable
2. Cultural norms	a) Physician’s end-of-life care attitudes, behaviors and decisions privy to cultural norms
	b) Death and dying perceived as a “taboo” topic in certain cultures
	c) Need for end-of-life care to be sensitive to different cultures encountered
3. Workplace cultural norms	a) Influencing views on death, end-of-life care attitudes, behavior and decision making
4. Societal expectations	a) Societal expectations promoting survival and death prevention
	b) Perception of treatment withdrawal as taking the life of one’s patient affecting physician’s end-of-life decision making
5. Legal standard	a) Fear of legal challenge affecting end-of-life care leading to defensive practice
	b) Unclear laws surrounding end-of-life practices breeding legal uncertainty
	c) Adherence to decisions despite perceived potential legal kickback
6. Professional Relationships	*Patients*
	a) Challenges faced during end-of-life communication
	b) Managing expectations of patients
	c) Inspiring interactions with patients
	*Patient’s family*
	d) Conflict between physician and patient’s family
	e) Effects of conflict on relationship
	f) Family members concerned for patient’s possible pain and distress
	g) Managing expectations of patient’s family members
	h) Family’s distress after end-of-life care discussion
	i) Empowering interactions with patient’s family members
	j) Factors affecting communication with family members
	k) Creation of soft landing when informing family about death
	l) Perception of intensive care unit as not conducive for palliative care discussions
	*Nurses & intensive care unit team*
	m) Support from other intensive care unit physicians to help manage end-of-life decisions
	*Physicians from other specialties*
	n) Challenges with interaction
	o) Lack of understanding of one another’s role
7. Professional Standards	a) Professional expectation for doctors to not cause death or harm to patients
	b) Responsibility to decide on withdrawal of treatment went against physician’s perceived professional standards
**Category/ Theme 5: Conflicts in providing end-of-life care**	
1. Interpretation of duty of the physician	a) Professional expectation that doctors should not cause death or harm to patients b) Responsibility of treatment withdrawal decision going against physician’s perceived professional standards c) Physician’s end-of-life care attitudes, behaviors and decisions d) Need for end-of-life care to be sensitive to different cultured encountered
2.Behavior of the physician	a) Doubts in self, conflicts in decision making b) Emotional and psychological overlay c) Internal conflict between beliefs and duties
3.Behavior of others	e) Conflict with intensive care unit nurses f) Challenges with interactions with other professionals g) Perception that nurses do not grasp the complexity of end-of-life decision making
4.Professional Standards	h) Conflict between respect for cultural norms and general practice i) Conflict between team members on how to interpret way to proceed in grey situations
**Category/ Theme 6: Coping strategies**	
1. Personal strategies	a) Effective communication to strengthen decision making position *Confidence:* b) Gaining confidence through experience c) Gaining confidence with end-of-life discussions d) Taking breaks from the intensive care unit or practicing on other sites
2. Strategies with patients	a) Collaboration with patient to reduce moral burden of decision making
3. Strategies with patient’s family	a) Creation of soft landing when informing patient’s family about death b) Collaboration with patient’s family to reduce moral burden of decision making
4. Strategies with colleagues	a) Conflict management interventions b) Emotional and experiential sharing of caring for dying patients c) Collaborations with interdisciplinary team members

##### Category/ theme 1: innate ring

Thnces an ICU physician’s views of their own mortality. It also includes their perspectives on whether or not they conceive the death of a patient as a failure. It includes the physician’s beliefs on their duty of care at end of life, particularly in relation to alleviating suffering, ensuring a ‘good death’ and their views surrounding one’s ‘right to die’.

##### Category/ theme 2: individual ring

This considers the physician’s beliefs, values, principles, personality, abilities, thoughts, emotions and actions with respect to death and dying. It includes factors that affect their thinking and decision making about EoL care such as the need to manage their own expectations, concerns and doubts about prognostication; their role in determining overall goals of care; and their ability to communicate these decisions to colleagues, patients and their families whilst showing empathy.

##### Category/ theme 3: relational ring

This considers the impact of the emotional and psychological self-doubt and apprehension they experience when caring for dying patients upon personal relationships the ICU physician deems important to them.

##### Category/ theme 4: societal ring

This considers the influence of sociocultural norms and societal expectations of the physician’s professional relationships and roles, and how they impact legal and professional standards. It includes the physical and practical issues affecting EoL care. It also considers the effect of caring for the dying on their relationships with colleagues, patients and their families.

##### Category/ theme 5: conflicts in providing EoL care

This moves beyond the general considerations discussed previously and regards specific accounts of internal conflicts affecting the physician, and conflicts with other healthcare professionals that arise in the provision of EoL care.

##### Category/theme 6: coping strategies

These include general and specific accounts of coping strategies adopted by physicians providing EoL care in ICUs. These accounts are categorized as a distinct category /theme despite their multimodal approach involving personal, professional and existential factors and strategies involving collaboration and sharing with other professionals.

## Stage 5 of SEBA: discussion

## Discussion

This SSR reveals an intricate array of intimately entwined responses by ICU physicians as a result of caring for dying patients. Highlighted by the themes ‘conflicts in providing EoL care’ and ‘coping strategies’, this SSR suggests that it has diverse ramifications including complex immediate and long-term effects on the ICU physician’s thinking, decision making and actions. Such change underscores the need to review how they are supported. The data rebuffs the notion that ‘single point interventions’ such as debrief sessions following a difficult case or a meeting with a psychologist or counsellor will sufficiently address the complex effects on their psycho-social, existential, personal and professional self-concepts. Rather this SSR will argue for a more longitudinal and holistic response to the support of ICU physicians that entails careful study of their individual concepts of personhood.

Contemplating these effects through the lens of the Ring Theory of Personhood (RToP) suggest that support should be directed at the beliefs, values and principles (Internal Constituents or IC) contained within the different rings of the ICU physician’s personhood. These arise from spiritual beliefs, familial credos, cultural norms, societal values and personal philosophies adopted. These, however, may surface in more than one ring thus reiterating their connection or entwined nature.

With ICs overlapping and also influencing a physician’s thinking, decision making and actions, ensuring that the ICs are in sync with one another is critical. For the rings to be in ‘synchrony’, these constituents must be congruous. For example, belief in the sanctity of life (Innate Ring) ought to be mirrored in how the physician perceives his role as a healthcare professional (Individual and Societal Ring) [30].

However, maintaining synchrony between the rings can be challenging particularly as ICU physicians face acute shortages of resources [31] and manpower, complex clinical cases [32] and unprecedented levels of death in ICUs. This is further aggrandized in times of national and global crisis [33]. These circumstances can create discordance within and between ICs through the creation of competing obligations. For example, when treatment options have been exhausted and continued ventilation of the ICU patient is deemed to be futile, the physician will be faced with the difficult choice of withdrawing ventilatory support and allowing the patient to die. This would run contrary to their belief in the sanctity of life [34, 35]. How the physician addresses this ‘catalyst’ will determine their actions.

A ‘catalyst’ is seen as a situation where a physician is forced to question their position and/or the ethical, moral, existential and personal beliefs, values and principles that they hold. If unaddressed a catalyst will result in ‘conflict’ within individual rings. If conflicts are not resolved effectively, they may result in further ‘dyssynchrony’ between rings as will be discussed later.

For now, this SSR suggests that the RToP provides a means of understanding the complex interplay of psycho-emotional, existential and relational issues at hand. The RToP suggests that in making decisions as to whether to withdraw futile treatment, physicians face catalysts in the form of doubts surrounding their roles in the provision of EoL care, the alleviation of suffering and their patient’s right to die [36, 37]. The moral, existential, personal and professional conflict between ICs in their Innate Ring is also made manifest in their Individual Ring as they face anxiety and doubts over their knowledge, skills and ability to provide effective and empathic care for these dying patients and their families as they meet their clinical obligations [38, 39].

These also impact their Relational Ring as they may compromise the quality and nature of personal relationships that the physician holds dear to them, thus threatening an important source of support as they grapple with these concerns [40]. Furthermore, struggles with balancing individual beliefs and values with societal, religious, cultural obligations and legal, professional, ethical standards of practice that stem from their Societal Ring serve to compound the ICU physician’s coping strategies and ultimately impair their ability to provide efficacious care [41–43]. The presence of multiple rings in conflict suggest that catalysts may generate ripples and have bearings on more than one.

Persistent unresolved conflicts between rings – the aforementioned dyssynchrony – may result in acute or prolonged moral, spiritual, emotional and psychological distress [44–46]. This may bear grave repercussions on their functioning as empathetic, reliable and effective healthcare providers with vulnerable patients under their care [47].

Conflict resolution may see ICU physicians prioritize their overarching goals based on their particular situation, professional and clinical obligations, beliefs, values and principles contained within their ICs and in keeping with regnant legal, professional and ethical standards [48, 49]. This process offers potential assurance and reprieve for the addled and overwhelmed physician. Yet in deciding to withdraw treatment, the physician’s decision to prioritize their clinical and professional obligations over their tendencies to err on the side of preserving life by virtue of their ICs in order to save the life of another patient with a better prognosis and less co-morbidities does not necessarily resolve the conflicts within the rings.

Here reframing the situation is critical. This process of reframing is guided by due consideration of contextual factors given the futility of prevailing treatment, the lack of alternative treatment options, the continued deterioration of the patient and the patient’s general poor prognosis [50–52]. Similarly, the physician may reframe the situation and acknowledge that it is also their duty to cease futile and potentially burdensome treatment to prevent suffering, avoid prolonging the dying phase or return the patient to the original disease trajectory after a trial of treatment [53–60]. This process of reframing is key to realigning ICs, attenuating conflicts within the rings and preventing or assuaging dyssynchrony between the rings. Resolution of individual conflicts must necessarily consider that the resolutions arrived at are not at odds with ICs in other rings so as to not rouse dyssynchrony.

Evidencing the need for a comprehensive response to conflicts is the ICU physician’s employment of multipronged coping mechanisms which draw on all four rings. This response includes a combination of timely, longitudinal, holistic interprofessional team support, collaborative efforts at care provision, refresher courses to gain greater confidence in one’s practice and time off/rotations out of the ICU as shown in Table 3. Resolving dyssynchrony helps to bring the rings into harmony or ‘synchrony’, facilitating the professional and personal growth of the physician [40].

Establishing ‘synchrony’ between the rings through use of prioritization and reframing also helps sustain the ICs against catalysts. This ability to bring about and sustain synchrony is seen as a sign of resilience.

### Resilience

Resilience is built by the ability to blunt or repel challenges to ICs brought about by catalysts. These take the form of internal and external factors (henceforth buffers). Internal buffers include prior experiences with similar catalysts and the possession of ethical, existential, practical and clinical skills [61–63]. Perhaps the most significant external buffer is discussing issues frankly with colleagues and garnering insight [64–67]. Other external buffers include having time outside of the ICU and a nurturing work environment [49, 68]. Buffers allow physicians “to enjoy a positive experience” caring for an end-of-life patient [37] despite the difficult circumstances and conflicts that may arise within their individual ICs.

**Fig. 4:**
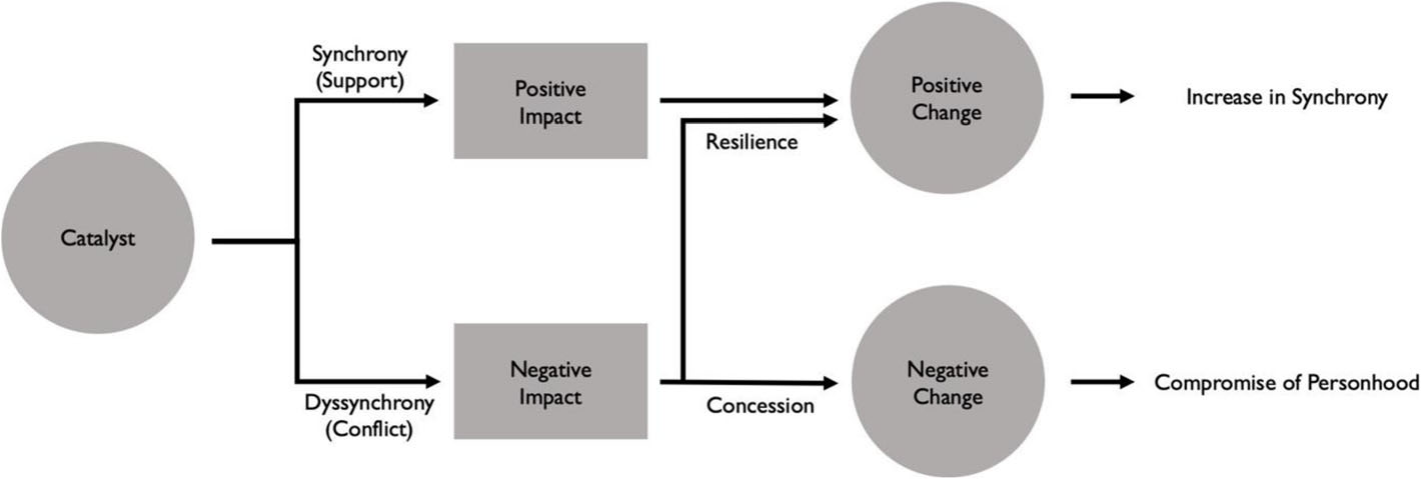
Resilience and Concession

Conversely ‘concession’ sees the individual in a state of prolonged dyssynchrony which manifests as negative emotional, psychological, and spiritual burden and in the long term is seen to result in burnout, disillusionment and exit from the specialty [69] (Fig. 4).

### Evolution of the ring structure

Whilst Radha Krishna and Alsuwaigh [28]‘s original concept of the RToP has served as a means of understanding the issues affecting ICU physicians, it could also prove useful as a means of guiding support for physicians.

**Fig. 5:**
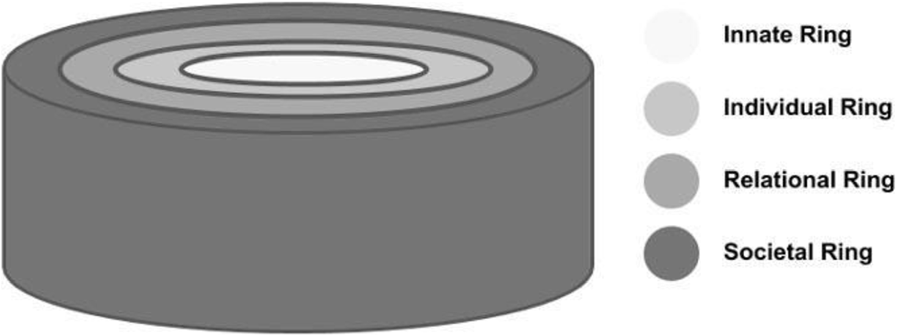
Cylinder Model

To operationalize the RToP, a review of its evolution is required. Developing from rings whose size depended on the number of elements within them, recent incarnations of the RToP conceive the rings as cylinders where the importance of ICs, as determined by the person, adds depth to the rings (Fig. 5). The concept of importance finds weight in this SSR and foregrounds the concept of prioritization and reframing. In keeping with this concept, the more important the element within the IC is in each ring, the closer it is to the inner aspect of the cylinder.

However, the data from this SSR adds a further dimension to this formulation. This third dimension comes from the impact of ‘buffers’ and the ability to maintain the rings in changing conditions. This dimension is consistent with the overarching goals of personhood which is to maintain the integrity of their individual concepts. The 3D perspective of the RToP serves as a reminder that concepts of personhood are highly personalized and involves input from the individual physician. Critically, the spheres may be employed as a means of holistically assessing the needs of ICU physicians in the care of dying patients.

**Fig. 6:**
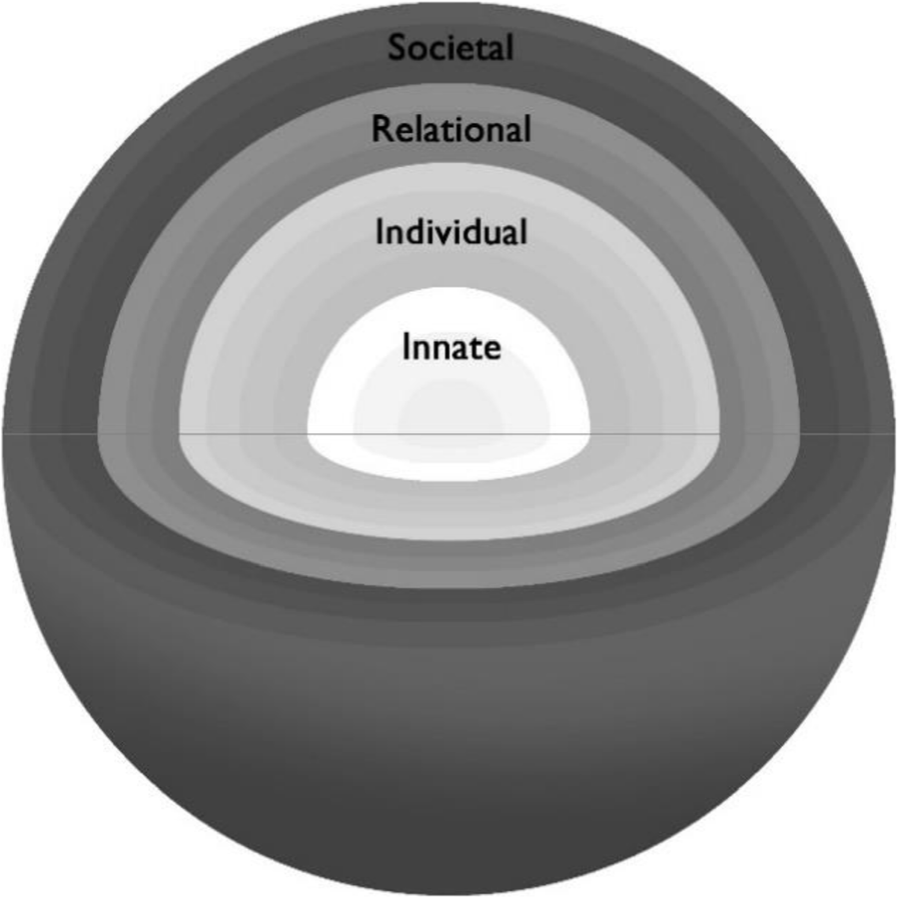
The Sphere Model

Here the size of the spheres is determined by the number of elements within the ICs and the width or thickness corresponds to the element’s importance arranged concentrically with the most important elements lying close to the core of this sphere. The depth of this sphere is afforded by the presence and quality of buffers. Here the quality of the buffers corresponds to their ability to repel catalysts and maintain the ICs. (Fig. 6).

## Limitations

Although the search process was vetted and overseen by the expert team, use of specific search terms and inclusion of only English language articles potentiates the possibility of key publications being omitted. In addition, whilst independent and concurrent use of thematic and content analysis by the team of researchers improved its trustworthiness through enhanced triangulation and transparency, biases cannot be entirely eradicated.

Use of the RToP in this context is also novel and the data captured in this SSR suggests that its use as a ‘tool’ to identify critical issues with physicians’ concepts of personhood still possesses limitations. Although the RToP provides a ‘snapshot’ of these prevailing concepts, the funneling process evidences the need for further studies to be conducted as to how personhood may evolve in both ordinary and extraordinary circumstances, and how physicians may address and cope with these diverse situational considerations. Finally, despite the presence of nursing data that echo the findings of this review, these findings may not truly reflect the unique challenges that ICU physicians experience, especially during pandemics such as COVID-19.

## Conclusion

The COVID-19 pandemic has presented unprecedented challenges for ICU physicians facing death and dying. Data drawn from ‘peace time’ suggests that ICU physicians cope variably with different issues they face, underlining the need for personalized understanding of their particular situation and the presence of comprehensive long term support.

Indeed, the RToP lends itself to the design of such support structures with the first step calling for identification and recognition of physicians who are in a state of ‘dyssynchrony’ or ‘concession’. Ethical guidance, intercollegial support and a nurturing work environment providing personalized debrief sessions and time out of the ICU will aid in reducing protracted dissonance between the beliefs, values and principles contained within each ring. This will naturally guide the physician towards ‘synchrony’ and alignment of these internal constituents – in short, the building of resilience.

As such, it is hoped that the four rings of the RToP will be carefully considered in the curation of longitudinal and holistic assessment tools used to discern the wide-ranging needs of overwhelmed ICU physicians. This is especially in the face of evidence that the impact of death and dying may ripple and implicate multiple rings of their personhood. It is only with assiduous analysis of all four rings that there can be greater certainty that all possible ramifications of death and dying are appropriately accounted for. These considerations if successfully attended to will certainly bolster the ICU physician.

Whilst these suggestions need further study, some of the lessons drawn are applicable to the COVID-19 pandemic where increased workload, manpower limitations, resource scarcity and complex triaging of vulnerable patients may see the amplification of moral and existential distress. Whilst resilience is crucial in ordinary ‘peace time’ as it wards against burnout, disillusionment and the phenomena which sees physicians leaving the specialty, it is especially necessary in the precarious climate of the COVID-19 era. With resilience not only do we protect the physician but we also protect the wider healthcare system.

## Supplementary information

Additional file 1. Pubmed Search Strategy. Search strategy employed as part of the systematic scoping review process. (DOCX 15.5 kb)

Additional file 2. Summary of included articles. Summaries of key points of articles included with MERSQI and COREQ quality assessment. (DOCX 131 kb)

Additional file 3. Summary of Thematic Analysis and Direct Content Analysis with References. Themes and categories identified in analysis with references. (DOCX 714 kb)

Additional file 4. Combined Themes with References. Combined themes after the funneling process with references. (DOCX 768 kb)

## Data Availability

All data generated or analysed during this study are included in this published article.

## Abbreviations

ICU: Intensive care units
SSR: Systematic scoping review
SEBA: Systematic evidenced based approach
RToP: Ring theory of personhood
PCC: Population, context and concept
MERSQI: Medical education research study quality instrument
COREQ: Consolidated criteria for reporting qualitative studies
BEME: Best evidence medical education
STORIES: structured approach to the reporting in healthcare education of evidence synthesis
EoL: End of life
IC: Internal constituent
