# Supplementary material for: The impact of caring for dying patients in intensive care units on a physician’s personhood: a systematic scoping review
Source: Philos Ethics Humanit Med. 2020 Nov 25;15:12. doi: 10.1186/s13010-020-00096-1 (PMC7685911; doi:10.1186/s13010-020-00096-1)
Supplement: Supplementary file 1 — Pubmed Search Strategy. Search strategy employed as part of the systematic scoping review process. (DOCX 15.5 kb) [file 13010_2020_96_MOESM1_ESM.docx]

*Additional file 1: PubMed Search Strategy*

|  |  | MeSH | Keyword |
| --- | --- | --- | --- |
| Population | **Doctors** | “Physicians” [Mesh] | Doctor [tiab] OR Doctors [tiab] OR intensivist [tiab] OR intensivists[tiab] OR resident [tiab] OR residents [tiab] OR physician[tiab] OR physicians[tiab] |
| Intervention / Exposure | Working with dying patients | "Terminally Ill" [Mesh] OR "Critical Illness" [Mesh] OR "Death" [Mesh] OR "Life Support Care" [Mesh] OR "Critical Care" [Mesh] OR "Palliative Care" [Mesh] OR “Clinical Deterioration” [Mesh] OR “Catastrophic Illness” [Mesh] | Dying [tiab] OR terminal [tiab] OR critical [tiab] OR intensive [tiab] OR life threatening [tiab] OR palliative [tiab] OR end of life [tiab] OR end-of-life [tiab] |
|  | in ICU | "Intensive Care Units"[Mesh] | neurocritical [tiab] OR neurointensive [tiab] OR Intensive treatment [tiab] |
| Comparison / control |  |  |  |
| Example outcome measures | Emotions  (positive and negative) | “Emotions” [Mesh] OR "Bereavement" [Mesh] OR "Anxiety" [Mesh] OR "Frustration" [Mesh] OR "Stress, Psychological" [Mesh] | grie* [tiab] OR sorrow* [tiab] OR mourn* [tiab] OR bereave* [tiab] OR  sad* [tiab] OR distress [tiab] OR stress [tiab] OR fatigue* [tiab] OR burnout [tiab] OR  emotion* [tiab] OR feel* [tiab] OR satisf* [tiab] OR fulfil* [tiab] |
|  | Leading to behaviour changes and behaviour mechanisms | “Stress, Psychological” [Mesh] OR “Adaptation, Psychological” [Mesh] OR “Attitude” [Mesh] OR “Behavior” [Mesh] OR “Defense Mechanisms” [Mesh] OR “Human Development” [Mesh] OR “Motivation” [Mesh] OR “Neurobehavioral Manifestations” [Mesh] OR “Personality” [Mesh] OR “Psychology, Social” [Mesh] OR “Affective Symptoms” [Mesh] | motiva*[tiab] OR incentiv*[tiab] OR attitude*[tiab] OR perspective* [tiab] OR approach*[tiab] OR behavio*[tiab] OR perform* [tiab] OR conduct* [tiab] |
|  | Biological / Physical impact  (positive and negative) | “Fatigue” [Mesh] OR “Body Weight” [Mesh] OR “Aging, Premature” [Mesh] OR “Asthenia” [Mesh] | energ* [tiab] OR revital* [tiab] OR reinvigorat* [tiab] OR fit* [tiab] or weak* [tiab] |

((((("Physicians" [Mesh] OR Doctor [tiab] OR Doctors [tiab] OR intensivist [tiab] OR intensivists[tiab] OR resident [tiab] OR residents [tiab] OR physician[tiab] OR physicians[tiab]))) AND (("Terminally Ill" [Mesh] OR "Critical Illness" [Mesh] OR "Death" [Mesh] OR "Life Support Care" [Mesh] OR "Critical Care" [Mesh] OR "Palliative Care" [Mesh] OR "Clinical Deterioration" [Mesh] OR "Catastrophic Illness" [Mesh] OR Dying [tiab] OR terminal [tiab] OR critical [tiab] OR intensive [tiab] OR life threatening [tiab] OR palliative [tiab] OR end of life [tiab] OR end-of-life [tiab]))) AND (("Intensive Care Units"[Mesh] OR neurocritical [tiab] OR neurointensive [tiab] OR Intensive treatment [tiab]))) AND (("Emotions" [Mesh] OR "Bereavement" [Mesh] OR "Anxiety" [Mesh] OR "Frustration" [Mesh] OR "Stress, Psychological" [Mesh] OR grie* [tiab] OR sorrow* [tiab] OR mourn* [tiab] OR bereave* [tiab] OR sad* [tiab] OR distress [tiab] OR stress [tiab] OR fatigue* [tiab] OR burnout [tiab] OR emotion* [tiab] OR feel* [tiab] OR satisf* [tiab] OR fulfil* [tiab] OR "Stress, Psychological" [Mesh] OR "Adaptation, Psychological" [Mesh] OR "Attitude" [Mesh] OR "Behavior" [Mesh] OR "Defense Mechanisms" [Mesh] OR "Human Development" [Mesh] OR "Motivation" [Mesh] OR "Neurobehavioral Manifestations" [Mesh] OR "Personality" [Mesh] OR "Psychology, Social" [Mesh] OR "Affective Symptoms" [Mesh] OR motiva*[tiab] OR incentiv*[tiab] OR attitude*[tiab] OR perspective* [tiab] OR approach*[tiab] OR behavio*[tiab] OR perform* [tiab] OR conduct* [tiab] OR "Fatigue" [Mesh] OR "Body Weight" [Mesh] OR "Aging, Premature" [Mesh] OR "Asthenia" [Mesh] OR energ* [tiab] OR revital* [tiab] OR reinvigorat* [tiab] OR fit* [tiab] or weak* [tiab]))
