# Supplementary material for: The impact of caring for dying patients in intensive care units on a physician’s personhood: a systematic scoping review
Source: Philos Ethics Humanit Med. 2020 Nov 25;15:12. doi: 10.1186/s13010-020-00096-1 (PMC7685911; doi:10.1186/s13010-020-00096-1)
Supplement: Supplementary file 2 — Summary of included articles. Summaries of key points of articles included with MERSQI and COREQ quality assessment. (DOCX 131 kb) [file 13010_2020_96_MOESM2_ESM.docx]

*Additional file 2: Summary of Included Articles*

| **Year of Publication**  **Author** | **Title** | **Background** | **Main empirical findings** | **Insights drawn** | **MERSQI Score** | **COREQ Score** |
| --- | --- | --- | --- | --- | --- | --- |
| K. Aita et al., 2010 | Physicians' psychosocial barriers to different modes of withdrawal of life support in critical care: A qualitative study in Japan | Despite a number of guidelines issued in Anglo-American countries over the past few decades for forgoing treatment stating that there is no ethically relevant difference between withholding and withdrawing life-sustaining treatments (LST), it is recognized that many healthcare professionals in Japan as well as some of their western counterparts do not agree with this statement. This research was conducted to investigate the barriers that prevent physicians from withdrawing specific LST in critical care settings, focusing mainly on the modes of withdrawal of LST, in what the authors believe was the first study of its kind anywhere in the world. | The main theme found in the paper suggest that the modes of withdrawal of LST are related to physicians’ psychosocial barriers that prevent them from withdrawing a certain type of LST, in particular mechanical ventilation.  Five subthemes emerged:   1. Differences among withdrawal of mechanical ventilation, PCPS, and artificial liver support- most informants did not choose to withdraw mechanical ventilation from dying patients 2. The length of time from withdrawal of life support till cardiac arrest- The most common reason cited by the informants regarding their unwillingness to withdraw mechanical ventilation was the immediate cardiac arrest of the patient following withdrawal, considering it a life-shortening act which causes distress for doctors. 3. Differences in the modes of withdrawal- Compared to other modes, mechanical ventilation withdrawal takes place when mechanical operation of the ventilator can be still be continued, bringing a psychological burden on physicians 4. Desire for a ‘‘soft landing’’-Most informants shared the view that the final phase of the patient’s life should be made peaceful by avoiding or easing drastic changes and soft landing is necessary for the family. 5. Paradoxical situation created by the desire for a soft landing- The desire for a soft landing coupled with the undesirable public views on withdrawal, has created a paradoxical situation in some cases, where case, the direct linkage between the withdrawal of the device and the patient’s death would not be evident is used to decide withdrawal instead of severity of condition. | The findings suggest what the Japanese physicians avoid is not what they call a life-shortening act but an act that would not lead to a soft landing, or a slow death that looks 'natural' in the eyes of those surrounding the patient.  This is to fulfill the psychosocial needs of the patient's family and the physicians, who emphasize on how death feels to those surrounding the patient.  The physicians’ perception of death and dying as a social relationship is likely to be one of cultural factors for the difference in end-of-life decision-making.  Unless withdrawing LST would lead to a soft landing, Japanese clinicians, are likely to feel that there is an ethically relevant difference between withholding and withdrawing LST. | NA | 17 |
| I. Almansour, J. Seymour and A. Aubeeluck, 2019 | Staff perception of obstacles and facilitators when providing end of life care in critical care units of two teaching hospitals: A survey design | One of the studies reviewed by Espinosa et al. (2008) designed a tool (The National Survey of Critical Care Nurses’ Perception of End-of-life Care) to measure American critical care nurses’ perceptions of obstacles and supportive behaviours in EoLC (Beckstrand and Kirchhoff, 2005). This paper reports a study that employed the survey of critical care nurses’ perception of EoLC in Jordanian critical care units.  Objective: To determine perceptions of Jordanian critical care staff about obstacles and facilitators to end-of-life care | The main theme of the paper is to identify obstacles to EoLC for physicians in terms of intensity (i.e. importance or magnitude) and frequency   1. Family members not understanding what life-saving measures really mean- Most intense obstacle and most frequent 2. Continuing treatments for a dying patient even though the treatments cause the patient pain or discomfort- 2nd most intense and 4th most frequent 3. Clinicians having to deal with angry family members- 3rd most intense 4. Family continually call the clinician wanting an update condition rather than calling the designated family member- 4th most intense and 3rd most frequent 5. Poor design of units which do not allow for privacy of dying patients or grieving family members- 5th most intense and 2nd most frequent 6. The unavailability of an ethics board or committee to review difficult patient cases- 6th most intense 7. Continuing intensive care for a patient with a poor prognosis because of the real or imagined threat of future legal action by the patient’s family- 5th most frequent   *Summaries only relevant to ICU Doctors | Insights into some of the mechanisms that may be behind our findings are provided by a qualitative study of physicians in the Middle East (Al-Awamer and Downar, 2014), which shows that it is the cultural norm in the Middle East for practitioners to avoid direct conversations about EoLC with relatives and to continue with interventionist treatment for as long as possible; this is partly because it is perceived that this is what the family desires but also due to lack of legal clarity, which leads to defensive practice.  There is a need to further explore the issues underlying perceptions about clinicians’ behaviours and family communication, which Jordanian staff perceived to be key barriers to quality EoLC and to find acceptable solutions to these that fit with Islamic culture. | 7.5 | NA |
| R. Amati and A. F. Hannawa, 2014 | Relational Dialectics Theory: Disentangling physician-perceived tensions of end-of-life communication | Several strategies have been suggested to enhance end-of-life interactions; however, a solid theoretical framework is needed for the development of effective systematic guidelines and interventions that can facilitate this goal. The present research study addresses this gap, choosing to focus particularly on the physician's perspective. It relies on Baxter and Montgomery's (1996) Relational Dialectics Theory to illuminate the complexity of reality doctors commonly face in interactions with their patients during end-of-life care. | All participants in this study identified Baxter and Montgomery’s (1996) contradictions in reflection on past end-of-life interactions with their patients. The following dialectical tensions and themes were identified.   1. Autonomy/connection- Having a connection with your patient while maintaining autonomy for yourself, to be able to make objective therapeutic decisions 2. Openness/closedness- Open yourself to better understand your patient while being closed to make objective decisions. 3. Novelty/predictability- Each case is different from the other, so it is novel. But on the other side, the certainty or the non-novelty lies in the fact that all of them belong to a schema of pathologies, so on the basis that you know what therapy you need to plan. 4. Standardization versus personalization- Feeling forced to adhere to standardization while facing an individual human being. Often tend to show frequencies and numbers in a mechanical way 5. Desire/ability- Desire to save their patients but at the same the ability to cure them was hindered by the reality of end-of-life   All participants suggested that dialectical tensions constantly emerge in the context of end-of-life communication  *Summaries only relevant to ICU Doctors | Dialectical tensions and contradictions need to be balanced to promote relational development and maintenance, which is of critical importance during end-of-life care for the physician, the patient, and the patient’s family members. The necessity for a human relationship that is built on mutualism emerges (see Roter & Hall, 1992), and advanced communication skills are required from physicians, patients, and families to optimize the interaction. Relational Dialectics Theory could be a valuable framework to facilitate this goal.  Also the contradictions might be levered if physicians learn how to communicate in a way that creates a mutual relationship with their patient. Specifically, physicians could be more effective in clarifying and managing the desire/ability tension.  The theoretical extensions that were facilitated in this study can be used as a framework to develop these and additional translational efforts toward promoting healthier and more satisfying end-of-life care through more competent communication. | NA | 16 |
| D. A. Asch, J. Hansen-Flaschen and P. N. Lanken, 1995 | Decisions to limit or continue life-sustaining treatment by critical care physicians in the United States: conflicts between physicians' practices and patients' wishes | The purpose of this study was to examine the withholding and withdrawing of life support by critical care physicians. We surveyed a national sample of 879 physicians practicing in adult intensive care units in the United States, in order to determine their practices with regard to limiting life-sustaining medical treatment, and particularly their decisions to continue or forgo life support without the consent or against the wishes of patients or surrogates. | Main findings are as follows   1. The vast majority of our respondents reported that they had withdrawn or withheld medical treatments from patients with the understanding that death would follow. 2. One third of our physicians reported withdrawing life-sustaining treatment from a patient not capable of making decisions and not represented by a surrogate decision maker. 3. Despite the growing acceptance of requests by patients or surrogates to limit medical treatment, a third of our physicians also reported refusing such requests.   Many physicians withhold or withdraw life-sustaining treatment unilaterally because they had judged that further intervention would be futile. | A third of our respondents have had to make decisions in the absence of a patient proxy identifies an extremely common problem, raises questions about how these decisions were made or evaluated, and suggests the need for guidelines to assist clinicians in these decisions.  Fears about malpractice litigation or perceptions of ethical or policy impediments, were also offered to explain why physicians might not limit treatment on request of patients or surrogates  There are no generally accepted rules of engagement for these conflicts, and the justification and appropriateness of our physicians' decisions is therefore unclear. In fact, it is doubtful that ethical principles regarding the appropriate practical definition of medical futility will ever be noncontroversial.  Arguments in favor of reducing the role of patients or surrogates in decisions relating to life-support may be excuses to avoid difficult but necessary discussions with patients, or used to disguise less defensible motives  Two distinct types of futility: Physiological futility and because further treatment is not expected to achieve a meaningful survival for the patient  Younger physicians are more likely than older physicians to report withdrawing mechanical ventilation. This finding may reflect a shift in behavior among recently trained physicians. | 6 | NA |
| R. A. Aslakson, R. Wyskiel, D. Shaeffer, M. Zyra, N. Ahuja, J. E. Nelson and P. J. Pronovost, 2010 | Surgical intensive care unit clinician estimates of the adequacy of communication regarding patient prognosis | Intensive care unit (ICU) patients and family members repeatedly note accurate and timely communication from health care providers to be crucial to high-quality ICU care. Practice guidelines recommend improving communication. However, few data, particularly in surgical ICUs, exist on health care provider opinions regarding whether communication is effective. This study aims to address this. | The main theme found in the paper suggest that among ICU nurses as well as ICU physicians and NPs, general dissatisfaction existed with how surgeons communicate prognosis, the opportunities to communicate with surgeons regarding prognosis, and whether the conversations were valued by the surgeon. Yet, surgeons reported good communication between themselves and nurses and ICU physicians and NPs.  Three subthemes emerged:   1. ICU daily goal sheets- Only ensures communication between ICU physician and ICU nurses, does not involve surgeons 2. The concept of “prognosis” may differ between health care provider groups- Surgeons emphasis is averting death and prognosis means keeping patient alive. ICU physicians and nurses emphasis is to avert suffering and prognosis means patients quality of life, hospital stay and future complications 3. Cultural environment- differences between surgical, nursing, and ICU physician/NP cultures–can also simplify, or complicate, discussions regarding prognosis   *Summaries only relevant to ICU Doctors | Aspects of surgical culture that could complicate the communication of prognosis include: the “rescue credo” –the need to “save” or “rescue” a patient from dying; that surgeons often associate patient death with personal failure and shame and professional “shame” if a patient does not receive all possible interventions.  Daily goal sheets for surgeons and ICU teams (nurses and/or ICU physicians and NPs) could direct the content of conversations as well as facilitate more frequent opportunities for discussions.  Routine multidisciplinary family meetings for ICU patients and patient families could further mitigate deficiencies highlighted by the surveys.  As palliative care teams become more prominent in the ICU, or as surgeons, ICU physicians and NPs, and ICU nurses become more skilled in palliative care, such discussions regarding prognosis may also become less problematic. | 7 | NA |
| E. Azoulay et al., 2009 | Prevalence and factors of intensive care unit conflicts: the conflicus study | RATIONALE: Many sources of conflict exist in intensive care units (ICUs). Few studies recorded the prevalence, characteristics, and risk factors for conflicts in ICUs. OBJECTIVES: To record the prevalence, characteristics, and risk factors for conflicts in ICUs. | In this large cross-sectional survey, we found that up to 70% of ICU staff members reported ICU conflicts. Intrateam disputes accounted for the majority of conflicts.  Two main themes regarding the conflicts in this study:   1. Staff meetings- conflicts were less likely to occur in ICUs that held regular staff meetings. Among general behaviours perceived as causing conflicts, the most common were personal animosity, mistrust, and poor communication within the ICU team. 2. End-of-life care- Cared for at least one patient who died within the last week, involved in premortem and post-mortem care of at least one dying patient within the last week. The main perceived sources of conflict related to end-of life care were lack of psychological support, absence of unit-level meetings, and problems with the decision-making process.   *Summaries only relevant to ICU Doctors | Ensuring that the same physician or nurse is not in charge of several dying patients at the same time might reduce conflicts  Explaining the principles of palliative care to families and having physicians and nurses work together to evaluate pain, anxiety, and other symptoms are simple means of decreasing conflicts while significantly improving the quality of death.  Suggest testing interventions designed to reduce conflicts, such as holding unit-level meetings at least once a week, and ensuring that each ICU staff member is responsible for no more than one dying patient at a time | 12 | NA |
| J. G. Baggs et al., 2012 | Who is attending? End-of-life decision making in the intensive care unit | PURPOSE: Traditional expectations of the single attending physician who manages a patient's care do not apply in today's intensive care units (ICUs). Although many physicians and other professionals have adapted to the complexity of multiple attendings, ICU patients and families often expect the traditional, single physician model, particularly at the time of end-of-life decision making (EoLDM). Our purpose was to examine the role of ICU attending physicians in different types of ICUs and the consequences of that role for clinicians, patients, and families in the context of EoLDM. | The attending is not an individual but a role that is held by different individuals sequentially over time or by multiple physicians at the same time. The overarching theme is that identifying who is acting in the ICU attending role for an ICU patient is difficult, frustrating for families and staff, and complicates EoLDM in particular. Surgeons are also unable to accept the impending deaths of their patients also contribute to delay.  The subthemes are:   1. ICU attending physicians- It was found to be the physician ultimately responsible for the patient. It was challenging to identify who was in the attending physician role for a given ICU patient at a particular time. There were differences by ICU and by type of patient concerning who might serve as an attending physician. 2. Multiple attending physicians- Multiple physicians could serve in the attending role simultaneously. 3. Rotations- Regardless of who qualified to be in the attending physician role, the role generally was temporary and was held sequentially by different physicians over time. 4. The ICU attending role in EoLDM- The ICU attending role around EoLDM varied by type of unit and patient. Medical intensivists generally were comfortable with discussion of limitation of treatment. Surgeons have difficulties acquiescing to the impending deaths of their patients and are less present, may result in delay in EoLDM 5. The ICU attending role and timing of EoLDM- Discussion of EoLDM generally happens earlier than in SICU. Surgeons rarely initiated treatment withdrawal discussions compared to physicians in MICU 6. The ICU attending role in EoLDM and nursing staff- Interactions between physicians and nursing staff in EoLDM varied by type of unit and type of patient. For medical patients, nurses felt more empowered to ask questions on rounds, about ‘‘ultimate goals of treatment’’ or about prognosis than for surgical patients. 7. The consequences of the ICU attending role for family involvement in EoLDM- Family members often were confused about who was in the attending role and become angry and frustrated by trying to participate effectively in EoLDM with multiple ‘‘attendings’’, resulting in deleterious effects on patient and family-centered care giving. | Surgeons’ difficulties acquiescing to the impending deaths of their patients, and confusion about which attending (surgeon versus intensivist) would assume responsibility for EoLDM led to delayed EoL discussions for surgical patients compared with medical patients.  Solutions lie in seeking creative changes in hospital systems of care to address the communication, coordination, and continuity problems generated by the complexities of attending roles in acute care, and especially around EoLDM in ICUs, including evaluation of the effects of these changes on families’ experience of care.  Palliative care consultation may offer improved communication, coordination, and continuity in working with families and patients in EoLDM. The effectiveness of their involvement will depend on their ability to negotiate the attending physician subcultures in EoLDM as well as deal with their own communication, coordination, and continuity issues on behalf of patients and families. Technical guidelines for ICU palliative care have been developed and should be of assistance  Requiring clinicians to examine how culture affects the system and structures within which they do our work and treat their patients and if need be call for cultural change. | NA | 17 |
| C. G. Ball et al., 2010 | The impact of country and culture on end-of-life care for injured patients: Results from an international survey | Up to 20% of all trauma patients admitted to an intensive care unit die from their injuries. End-of-life decision making is a variable process that involves prognosis, predicted functional outcomes, personal beliefs, institutional resources, societal norms, and clinician experience. The goal of this study was to better understand end-of-life processes after major injury by comparing clinician viewpoints from various countries and cultures. | The main theme of the paper is that physician’s beliefs regarding end of- life care vary across country and geographic region as the result of a complex interaction between societal norms, religion, and technology.  Subthemes:   1. Guidelines and law- Although most clinicians thought that end-of-life decision making varied significantly across countries and cultures, few respondents had formal medical futility laws or guidelines (instituted by local governmental bodies) to direct their Practice 2. Religion- most clinicians denied that their own individual faith influenced the end-of life care they provided to injured patients 3. Work experience- most respondents also stated their end-of-life care had changed with greater clinical experience 4. Clinician role differences within the ICU amongst countries- In most regions, the attending critical care physician directed EoL care. In the United States, most were made by the admitting trauma surgeon 5. Ethical services- Asia and Europe were particularly underrepresented amongst institutions that offer ethical services. Interpreted utility ranged widely, with Europe, Asia, and Australia finding them to be particularly unhelpful 6. Resource limitations- Majority of respondents in both Asia and South Africa believed that resource limitations influenced their decisions 7. Age and physical dysfunction- Regardless of the region, most clinicians responded that patient age was a significant factor in end-of-life decision making for patients. Most respondents believed that a particular level of dysfunction altered their decision making such as the function of the patient’s diaphragm | Although study respondents that physician’s beliefs regarding end of- life care vary across country and geographic region as the result of a complex interaction between societal norms, religion, and technology, most clinicians denied that their own individual faith influenced the end-of life care they provided to injured patients. This contradicts the preliminary findings of a large European study that detected significant differences based on both doctor and patient religion  The concept of distributive justice affected EoL decisions in South African respondents. However, despite ample resources, US clinicians were much more reluctant to suggest withdrawing interventions on patients. This viewpoint may result from a complex interaction between the Western hemisphere’s heavy reliance on patient autonomy, litigious concerns, and the nonseparation of trauma surgeon and intensivist.  Perhaps, there is an inherent emotional conflict between the trauma surgeon focusing on using aggressive therapies to prolong life and the intensivist directing family discussions and end-of-life decisions for injured patients | 11 | NA |
| A. E. Barnato, J. A. Tate, K. L. Rodriguez, S. L. Zickmund and R. M. Arnold, 2012 | Norms of decision making in the ICU: a case study of two academic medical centers at the extremes of end-of-life treatment intensity | Differences in use and withdrawal of life-sustaining treatments (LSTs) in the intensive care unit (ICU) likely contribute to variations in end of- life treatment intensity between similar academic medical centers (AMCs), yet little is known about the norms of decision making underlying these differences.  PURPOSE: To explore norms of decision making regarding life-sustaining treatments (LSTs) at two academic medical centers (AMCs) that contribute to their opposite extremes of end-of-life ICU use. | The main theme of the paper is at the LI-AMC (Low-intensity AMC), LST was a means to an end whereas at the HI-AMC (High intensity AMC), it was an end in itself. The differences in decision making largely boiled down to the differences in the subthemes.  Subthemes:   1. Goals of life-sustaining treatment- At the LI-AMC, LST was the means to an end (recovery). At the HI-AMC, The goals of LST were to meet narrow physiologic objectives or avert death in the ICU 2. Determination of dying- At the LI-AMC, many patients were perceived as dying and there was seldom disagreement among team members or consultants. At the HI-AMC, there was often disagreement between services and even ambivalence on the part of individual critical care attendings regarding the determination of dying 3. Harms of commission versus omission- At the LI-AMC there was a particular focus on avoiding harms of commission. At the HI-AMC, harms of omission loomed larger 4. Physician self-efficacy for LST decision making- At the LI-AMC, Critical care physicians have a high degree of self-efficacy for decision making regarding life-sustaining treatment. They view family requests for continued treatment as part of the normal trajectory. At the HI-AMC Critical care physicians externalize the locus of control for decision making to patients, families, and specialists who they believe expect aggressive treatment. They view family requests for continued treatment as a mandate. 5. Origins of norms- The origin of the interest in end-of-life decision making at the LI-AMC was attributed to an influential internist and ethicist, homogeneous approach is promoted by retention of trainees as faculty, strong social norms protected against countervailing influences. The origin of the approach to end-of-life decision making at the HI-AMC was attributed to the institution’s status as a referral center attracting patients expecting treatment other centers would not provide such as transplants, sunk costs motivated continued investment and norms of treatment for complex referral populations created spillover effects for typical admissions   *Summaries only relevant to ICU Doctors | Variation in approach to end-of-life decision making was minimized by hiring faculty who also trained at the AMC, among whom the norms had been internalized as values  Withdrawal of LST, which was rare, appeared to be based on ‘‘physiologic futility’’ in the face of inexorable deterioration despite maximal LST, because critical care physicians and specialists did not agree that the patient was dying before that. This did not manifest as open conflict, but instead frustrated passivity on the part of critical care providers embodied by frequent complaint about specialist decision making, suggesting a ‘‘learned helplessness’’ based on prior reprisals. | NA | 19 |
| M. D. Barnett, B. R. Williams and R. O. Tucker, 2016 | Sudden Advanced Illness: An Emerging Concept Among Palliative Care and Surgical Critical Care Physicians | BACKGROUND: End-of-life discussions in critically-ill patients with acute surgical conditions may be rushed and occur earlier during hospitalization. This study explores the concept of sudden advanced illness (SAI) and its relevance to patients requiring Palliative and Surgical Critical Care. | The study explored the definitional components of SAI and also the difficult decision-making and emotions involved.   1. Definitional Components of SAI- Sudden and unexpected and typically in healthier and younger patients 2. Uncertainty in prognostication- Making it difficult to discuss prognosis for doctors and for families to know what to expect and potentially engendering in them a false sense of hope 3. Shift in decision-making responsibility- Doctors have to discuss end of life decisions with families who are catapulted into surrogate decision-making roles often without prior discussion of end-of-life preferences. 4. Overwhelming nature of surrogacy- the onset of an acute condition often comes as a shock to family members. Patient are typically also incapacitated and unable to participate openly in discussions of end-of-life goals. Such discussions can be highly emotionally charged for family members who typically are not prepared for dealing with life and death issues. This compounds the decision-making process and exacerbates the stress 5. Comfort Level With SAI- Physicians reported initial discomfort with SAI, a discomfort that is less acute over time 6. Comfort with family discussions- For some surgeons, there was a discomfort with family discussions and prognosis, such that discussions of end-of-life goals were best left to someone else 7. Emotional challenge of SAI- SAI is emotionally charged and often intensely personal, reflecting the age and life stage of many of the physicians themselves. | The stress of caring for patients with SAI is managed through coping skills developed on the job over many years of experience. However, coping strategies cannot fully moderate the cumulative emotional impact of working with patients in life and death situations.  Sudden advanced illness is more difficult for all of those involved, including physicians and other staff. Formal care providers are forced into discussions of sensitive end-of-life issues often without any prior relationship with the patients. Physicians report being confronted by their own mortality as these younger patients are often similar in age and stage of life. All agreed that the grief encountered in these cases was much more intense.  The emerging concept of SAI is an important one for understanding how the palliative medicine consultation process can enhance the care plan process. Further study of the potential benefits of palliative medicine integration into the ICU as it relates to complicated bereavement, family satisfaction, trainee education, provider satisfaction, and communication with families will be crucial in the future. | NA | 16 |
| E. Barton, 2007 | Situating end-of-life decision making in a hybrid ethical frame | Good communication in EoL (end-of-life) discussions is described at a general level in the literature, but there are few studies of EoL discussions at the level of interaction, with data drawn from the actual talk between physicians and families. In this article I present a discourse analysis of EoL discussions from an American ICU (intensive care unit) where the decision to withdraw life support is situated in a hybrid ethical frame co-constructed as the final phase of the EoL discussion. Using Mishler’s (1984) well-known distinction between the voice of medicine and the voice of the lifeworld, I show how both of these frames are drawn upon in the joint interactional construction of a hybrid ethical frame. | The main finding is that Phase 4 of the end-of-life discussion is a hybrid discourse that merges the voice of medicine and the voice of the lifeworld. Both physicians and families do interactional work to bring matters of importance to the discourse as topics, with repetition within and across the discussions indicating the importance of particular topics for both physicians and families   1. Physician-initiated topics    1. What physicians repeat and thereby emphasize in their Phase 4 reviews of the end-of-life decision is its medical appropriateness, situating the decision as an ethical one    2. Physician also draws upon a lifeworld frame by defining death in ordinary language and a cliche´. With a combination of medical–lay language, this Phase 4 discussion thus constructs both the voice of medicine and the voice of the lifeworld      1. Family-initiated topics to physicians    1. Families share an interest in the medical topic of process    2. Families, too, raised the topic of reviewing the EoL decision, primarily by drawing upon the lifeworld.    3. The topic of consciousness also was initiated by families    4. In these exchanges and when addressing concerns, physicians address the families’ concerns within the frame of medicine. | A physician draws upon Medical Ethics where a decision to withdraw life support is an ethical one if and only if it is a case of medical futility. Families’ frame of the ethics of death is thus drawn from ethics of personhood, the standard of death defined as the loss of human consciousness. The hybrid term “right” figures functions not only to index accountability to the definition of death in the medical and lifeworld frames, but also to index the decision as one within a shared ethical consensus. This analysis has implications for conducting the end of the EoL discussion, both in terms of content and interaction.  More specifically, this work suggests that physicians should not be surprised at the amount of repetition that goes on in the EoL discussion after the decision has been made. This repetition may be crucially functional in establishing families’ satisfaction that the decision is an ethical one.  The topic of consciousness emerges as interactionally marked for both families and physicians in the end-of-life discussion, though a difficult topic for physicians to address, it is vital to address in the EoL decision making. Physicians may be required to address rather than finesse the topic of death as the loss of human consciousness, not necessarily within their own ethical frame that demands medical certainty but within the family’s ethical frame that requires verification that the person’s human consciousness is lost. This reassurance may be critical in allowing families to situate their decision as an ethical one | NA | 8 |
| E. Barton, M. Aldridge, T. Trimble and J. Vidovic, 2005 | Structure and variation in end-of-life discussions in the Surgical Intensive Care Unit | The research reported here is an exploratory discourse analysis of a corpus of six end-of-life discussions in a Surgical Intensive Care Unit (SICU), describing the structure and variations of the four phases of an end-of-life discussion in terms of the function of each of these phases: the Opening (Phase 1), Description of Current Status (Phase 2), Holistic Decision Making (Phase 3), and Logistics of Dying (Phase 4). Of particular interest is Phase 2, in which the presentation of medical information culminates in an inferential summary statement that functions to establish the patient’s status as terminal. We argue that it is Phase 2 that is crucial in the functional progression of an end-of-life discussion toward a decision to move from therapeutic to palliative care. | The paper found that the end-of-life discussion includes four phases, which were titled with respect to their functions:   1. Opening (Phase 1)- function of the Phase 1 Opening is to establish the traditional role structure of a medical encounter 2. Description of Current Status (Phase 2), - the function of the Phase 2 Description of Current Status is to establish the patient’s status as terminal 3. Holistic Decision Making (Phase 3)- the function of the Phase 3 Holistic Decision Making is to achieve a consensual decision to withdraw life support 4. Logistics of Dying (Phase 4)- function of the Phase 4 Logistics of Dying is to develop an elaborated description of the pending death of the patient in a combined medical– lay discourse of palliative care. | The teaching literature recommends the ideal of a ‘two-way’ discussion, but the discourse practices of physicians, as well as the interactional contributions of family members, encourage, or even force, a traditionally asymmetrical relationship between the physician and the family.  In a busy practice setting, may be more efficient to practitioners to use monologues despite recommendations to practice patient-centered communication with questions and dialogue  The research also points to the functional mix of ordinary and medical language in end-of-life discussions: coming to a consensus about terminal status and making a decision to withdraw life support took place in ordinary language, but the structure of an end-of-life discussion also includes considerable attention to the specification of this decision to move to comfort care in the combined medical–lay discourse of Phase 4 | NA | 10 |
| S. Beck, A. Loo and S. Reiter-Theil, 2008 | A "little bit illegal"? Withholding and withdrawing of mechanical ventilation in the eyes of German intensive care physicians | Research questions and background: This study explores a highly controversial issue of medical care in Germany: the decision to withhold or withdraw mechanical ventilation in critically ill patients. It analyzes difficulties in making these decisions and the physicians' uncertainty in understanding the German terminology of Sterbehilfe, which is used in the context of treatment limitation. Used in everyday language, the word Sterbehilfe carries connotations such as helping the patient in the dying process or helping the patient to enter the dying process. Yet, in the legal and ethical discourse Sterbehilfe indicates several concepts: (1) treatment limitation, i.e., withholding or withdrawing life-sustaining treatment (passive Sterbehilfe), (2) the use of medication for symptom control while taking into account the risk of hastening the patient's death (indirekte Sterbehilfe), and (3) measures to deliberately terminate the patient's life (aktive Sterbehilfe). The terminology of Sterbehilfe has been criticized for being too complex and misleading, particularly for practical purposes. | 1. Interpreting the patient’s wishes and best interest    1. The physicians seemed to have specific difficulty in making the decisions of treatment limitation.    2. Physicians tend to have a paternalistic attitude, claims to interpret a patient’s interest better than the patient him/herself had been able to do before knowing the resulting medical situation 2. Withdrawal versus withholding ventilation    1. Physicians find it more difficult to withdraw rather than withhold treatment.    2. Judgments of withdrawal are emotionally and subjectively shaped as some physicians expressed their inability to formulate ‘‘reasonable’’ arguments for their judgments and decisions.    3. Legal uncertainty about legitimacy of decisions 3. Understanding the terminology of Sterbehilfe    1. Erroneous understanding by some physicians    2. Would decide for and defend a course of action that they themselves think is prohibited by law. 4. Perception of support for decision-making    1. Most of the physicians agreed that guidelines on end-of-life care such as those of the German Medical Association are helpful.    2. Clinical ethics case consultations only in a few institutions.    3. Some felt that the application of general rules to individual cases is problematic    4. Some did not know the content of the guidelines | In the German ethical and legal debate, previously articulated patient wishes are largely considered as ‘‘presumed patient wishes’’ in the context of an actual situation. They are thus treated as indications rather than explicit and authentic patient wishes.    The use of non-invasive ventilation as an alternative is a practical approach that provides the best possible chance to allow the patient to regain consciousness, and to keep him/her alive until his/her wishes become known  There is significant uncertainty about the legal classification of the withdrawal of mechanical ventilation. This can cause significant ethical and emotional burden to physicians as well as contribute to the continuation of meaningless treatment, and to the prolongation of the patient’s suffering.  Confusions on ethics and law create irritation and anxiety rather than intellectual stimulation in clinical decision-making. This calls for efforts towards more consistent practice rules and guidelines that are easily applicable then current ones. Furthermore, these situations are particularly burdensome and troubling for the clinicians, esp. for those who would decide for actions they (erroneously) consider illegal.  Ethics education has to continue during clinical training in order to create better opportunities for the transfer of conceptual knowledge to practical situations.  A specific need for orientation and support among intensive care physicians regarding their ethical and legal competence with practical cases in the ICUs.  Physicians tend to make decisions that are emotion-guided and based on fear of legal prosecution. This could and should be addressed by a more open discussion of these issues as well as by systematically providing information on the permissible and impermissible forms of treatment limitation or end-of-life care.  The reluctance of physicians to discuss these issues freely in their institutions is sometimes attributed to Germany’s historical background, and the fear of being misunderstood as invoking ‘‘euthanasia’’ in the sense of Nazi medicine. At the same time, discussing issues of dying in high-tech medicine and society touches a modern taboo. | NA | 17 |
| L. A. Brooks, M. J. Bloomer and E. Manias, 2019 | Culturally sensitive communication at the end-of-life in the intensive care unit: A systematic review | The objectives of this systematic review were the following: (i) to describe whether culturally sensitive communication is used by clinicians (nurses and physicians) when communicating with patients and families at the end-of-life in the intensive care unit and (ii) to evaluate the impact of culturally sensitive communication at the end-of-life. The systematic review question was how is culturally sensitive communication used by clinicians when communicating with patients and families at the end-of-life in the intensive care unit? | Two major themes emerged: communication barriers and cultural and personal influences on culturally sensitive communication.   1. Communication barriers- Communication barriers were identified in eight studies, influencing the timing and quality of culturally sensitive communication at the end-of life. 3 subthemes:    1. Clinician roles in communication- Physicians were seen to have a central or leading role, including coordinating communication with families including conveying prognosis and seeking consensus on resuscitation decisions; however, multiple contributing factors affected the ability of physicians to communicate with families with cultural sensitivity    2. Communication challenges- Shift changes and the rotation of staff in the ICU were identified as impacting clinicians, limiting their ability to build rapport with patients and families, and leading to ineffective communication among clinicians, patients, and families, such as disagreement regarding the prognosis, treatment plan, and end-of life care    3. Knowledge deficit- physicians may have a knowledge deficit related to the use of culturally sensitive language, leading to rushed conversations, false hope, and the use of language that families may not understand 2. Cultural and personal influences on communication- Cultural and personal influences on communication at the end-of-life was present in eight studies. 3 subthemes:    1. Cultural influences on communication- When cultural and religious preferences were not identified and accommodated in care and communication, conflict sometimes occurred between clinicians, and patients and families. Families also lacked awareness of how to communicate their cultural needs with clinicians, further increasing the risk of conflict    2. Clinicians' personal and sociocultural characteristics- physicians' approaches to end-of-life communication differ according to their own religious affiliation    3. Patients' and families' ethnic, cultural, and religious backgrounds- People from some cultures or religion may not fully understand the concepts of end-of-life care, leading to potential confusion and conflict. Some also had optimistic, unrealistic expectations, resulting in conflict and issues in exchange of medical information   *Summaries only relevant to ICU Doctors | Some physicians avoided conversations with families, assuming that they would not understand the prognosis or that condoning end-of-life care may not be acceptable  Using professional interpreters and encouraging patients and families to participate in communication regarding prognosis and end-of-life care are other strategies recommended in research evidence.  implementing appropriate healthcare organisational structures and policies to support culturally sensitive communication education  The cultural and personal characteristics of clinicians, patients, and families and how each of these characteristics influences the quality of culturally sensitive communication at the end-of-life are also highlighted in this systematic review | NA (AMSTAR: Low quality review) | NA (AMSTAR: Low quality review) |
| L. A. Brooks, E. Manias and P. Nicholson, 2017 | Barriers, enablers and challenges to initiating end-of-life care in an Australian intensive care unit context | BACKGROUND: Patients admitted to Australian intensive care units are often critically unwell, and present the challenge of increasing mortality due to an ageing population. Several of these patients have terminal conditions, requiring withdrawal of active treatment and commencement of end-of-life (EoL) care. OBJECTIVES: The aim of the study was to explore the perspectives and experiences of physicians and nurses providing EoL care in the ICU. In particular, perceived barriers, enablers and challenges to providing EoL care were examined. | The study reports 3 main themes in EoL care: Barriers, Enablers and Challenges  Barriers   1. Conflict between intensive care clinicians and external medical teams- The physician participants discussed the challenge of reaching medical consensus about the futility of actively treating a patient 2. Availability of education and training- physicians felt that limited opportunities existed in learning about EoL communication during their medical training 3. Environmental limitations- intensive care environment not conducive for EoL care and discussions   Enablers   1. Collaboration- Good collaboration amongst all parties facilitates good EoL care. Gaps were also identified regarding complexities in collaborating to achieve consensus among the healthcare team and families 2. Leadership during transitions of care- Medical leadership was emphasised as an important facilitator in transitions of care   Challenges   1. Difficulties surrounding communication and decision making- Language barriers, poor communication and clinician inexperience in conducting difficult conversations 2. Expectations of the family- Unrealistic family expectations, different cultural aspects and roles in families that affect how they react to situations, difficulty understanding the situation.   *Summaries only relevant to ICU Doctors | Physician participants discussed that the level of conflict may increase with surgical teams  There is a gap in the current education delivered to physicians in communicating with patients and their families at EoL. There was a lack of education and training opportunities  A structured education program, individually tailored to nurses and physicians, should be developed to provide all clinicians who engage in EoL care discussions with appropriate knowledge and skills.  The results of this study reinforce current evidence that decisions regarding EoL care are not collaborative  To facilitate a more consistent approach EoL care leaders of nursing and medical backgrounds, and patient support coordinators, are needed in ICU to encourage clinicians to communicate with other clinicians, and with family members about plans for EoL care | NA | 19 |
| L. A. Brooks, E. Manias and P. Nicholson, 2017 | Communication and Decision-Making About End-of-Life Care in the Intensive Care Unit | BACKGROUND: Clinicians in the intensive care unit commonly face decisions involving withholding or withdrawing life-sustaining therapy, which present many clinical and ethical challenges. Communication and shared decision-making are key aspects relating to the transition from active treatment to end-of-life care. OBJECTIVES: To explore the experiences and perspectives of nurses and physicians when initiating end-of-life care in the intensive care unit. | Communication   1. Timing of EoL Care Discussions- Poor timing of EoL care discussions was associated with patient and family distress, and discomfort for health care professionals directly involved with patient care. 2. Difficult Conversations- Difficult conversations and inexperience in conducting difficult conversations were viewed as a major challenge by physicians.   Shared Decision-Making   1. EoL Care Plans- physician participants perceived the EoL care plan to be a useful tool that formalized what was often discussed but not documented. The physicians also observed that the plan provides a structure that clearly identifies responsibilities, serving as a reminder that there were certain aspects of care that needed to be attended to following the commencement of the EoL care plan. 2. Multidisciplinary Acceptance of EoL Care Plans- The difficulties of reaching multidisciplinary acceptance of the EoL care plan were frequently discussed 3. Collaborative Decisions Involving Patients and Families- decisions to apply treatment limitations, and decisions to initiate EoL care.   *Summaries only relevant to ICU Doctors | Despite extensive focus in practice on initiating and delivering EoL care, conflict between health care teams often contributed to poor timing of EoL care discussions, affecting timely commencement of EoL care plans  Physicians both recognized that EoL discussions are occurring too late in a patient’s hospital admission and that further work is required to improve the communication skills of clinicians and collaboration between health care teams to promote early EoL conversations  Implications associated with lack of multidisciplinary acceptance of EoL care plans include prolongation of dying related to missed opportunities to provide optimal EoL care and moral and emotional distress among patients, families, and health care providers  In Australia, shared decision-making is recommended when developing EoL care plans; however, the views and wishes of the patient and family are relevant but not determinative in decision-making based on the patient’s best interests. This situation poses many ethical challenges for health care staff, particularly when there are complex family dynamics and unrealistic family expectations  Clear organizational processes with a collaborative decision-making framework must be in place to facilitate these leadership roles in establishing early EoL care plans where appropriate. | NA | 21 |
| T. Cardoso, T. Fonseca, S. Pereira and L. Lencastre, 2003 | Life-sustaining treatment decisions in Portuguese intensive care units: a national survey of intensive care physicians | INTRODUCTION: The objective of the present study was to evaluate the opinion of Portuguese intensive care physicians regarding 'do-not-resuscitate' (DNR) orders and decisions to withhold/withdraw treatment. | These are the main result findings:   1. The most important criterion for DNR orders, and decisions to withhold or withdraw treatment was the probability of survival from the acute episode, followed by the patient’s wishes 2. No physicians considered age of the patient to be the most important criteria for arriving at end-of-life decisions. 3. The number of physicians who felt that DNR orders should be discussed with patients and/or their relatives was three times greater than the number who actually do it 4. Only 12–15% of the respondents stated that they would involve nursing staff in these decisions. 5. Of respondents in the present study, 4.6% and 7.4% indicated that the doctor on duty is the sole decision maker regarding withholding treatment and DNR orders, respectively. Of those who responded, 4% felt that this situation is appropriate 6. Documentation of decisions is poor | Giving one person the power to make life and death decisions is dangerous, and the responsibility is a heavy one.  Even when clinicians make decisions with the best evidence available, their own ethical, social, moral and religious beliefs can influence these decisions. In our survey we found that sex, years of professional experience and religion influenced the way in which questions were answered. | 8.5 | NA |
| E. Chen, J. J. McCann and O. B. Lateef, 2015 | Attitudes Toward and Experiences in End-of-life Care Education in the Intensive Care Unit: A Survey of Resident Physicians | INTRODUCTION: Resident physicians provide the most physician care to intensive care unit (ICU) patients. The body of literature about residents' palliative and end-of-life care (PC/EoLC) experiences in the ICU is limited. To our knowledge, this is the first study to assess resident physicians in multiple specialties regarding PC/EoLC in the ICU. | 1. Residents’ Opinions and Attitudes Toward PC/EoLC- Overall, residents felt somewhat prepared to care for critically ill patients at the EoL. They were only moderately comfortable dealing with EoL issues and with the training they received 2. Comfort With Family Meetings- residency program and ICU months completed were significant predictors of comfort with family meetings 3. Technical Skill- residency program and importance of religion were significant predictors of technical skill 4. Education on PC/EoLC- females having significantly more positive views about the importance of education on PC/EoLC in the ICU setting compared to males. Residents who had pronounced more dying patients had significantly more positive views about the importance of education on PC/EoLC in the ICU setting 5. Total Measure- ICU months completed and importance of religion were significant predictors of scores on the total measure, with fewer months completed and higher importance of religion associated with more negative opinions and attitudes | In addition to encouraging role modelling of PC/EoLC skills and improving PC/EoLC education at all stages of training, increasing PC/EoLC encounters should be recommended to residents to help them increase their comfort and skill through experience.  Efforts should be made to increase PC/EoLC teaching throughout the medical education system and to provide feedback to residents on their PC/EoLC skills in the ICU. Future directions could include educating critical care faculty in providing feedback to residents and creating a specialized ICU PC/EoLC education module, ideally with objective studies to measure effectiveness | 6.5 | NA |
| N. Chikhladze et al., 2016 | Mismatch between physicians and family members views on communications about patients with chronic incurable diseases receiving care in critical and intensive care settings in Georgia: a quantitative observational survey | Background: Physicians working in critical and intensive care settings encounter death of chronic incurable patients on a daily basis; however they have scant skills on how to communicate with the patients and their family members. The aim of the present survey is to examine communication of critical and intensive care physicians with patients' family members receiving treatment due to chronic incurable diseases/conditions and to compare the views of families with physicians working in critical and intensive care settings. | Comparison of physicians’ and patients’ family members” responses to paired questions showed substantial differences. Physicians have a better perception of frequency and quality of the following topics compared to families:   1. Possible deterioration of a patient’s condition 2. How patient death may occur 3. Future treatment plans 4. Life related topics associated and important to the patient 5. Respect to patients’ spiritual attitudes and religiousness 6. Overall satisfaction with communication   The significant contrast in responses to paired questions highlights the problems in communication between CIC physicians and family members of patients dying from chronic incurable diseases | Some of this difference may reflect the state of stress of the patients’ family members when they receive bad news about the prognosis. It can affect recalling of the information given  Medical staff addressed non-medical aspects such as patients’ family members’ religious and spiritual beliefs with restraint and/or hesitation. This may be one of the main causes of patients’ family members dissatisfaction by communication with CIC physicians. It appears physicians have the appropriate attitudes, just their knowledge and skills need improvement  It is revealed that CIC physicians talk about patient death more frequently with patients’ family members, than with the patients. On the one hand, this reflects the fact that frequently it is difficult or impossible to communicate with the patients due to their altered consciousness. On the other hand, this fact might be explained by deficiency of relevant communication skills in “breaking bad news” related with the gap in professional education—on all levels of specialization  Physicians find it difficult to switch abruptly from the hope to survive and cure to preparation for dying and care at the end-of-life. Such transformation of medical care represents one of the most challenging steps in CIC settings  The persistence of the tradition of withholding the “bad diagnosis”, as well as “stigma” and “taboo” associated with terminal illnesses in Georgia, further aggravates the problem  Development of special training and qualification courses in palliative care for CIC physicians emerged as the urgent goal | 10 | NA |
| N. Çobanoglu and L. Algier, 2004 | A qualitative analysis of ethical problems experienced by physicians and nurses in intensive care units in Turkey | Conflict associated with decisions about withholding or withdrawing life-sustaining treatment may negatively affect patient care and the satisfaction of health care providers and family members. The objective was to suggest techniques to prevent potential conflict in solving ethical problems between physicians and nurses in intensive care units.  In this qualitative study, we aimed to identify and compare the ethical problems perceived by physicians and nurses in intensive care units at Baskent University hospitals in Turkey. | Physicians believed End-of-life decisions was the most common problem, followed by Communication and Hierarchical problems then Social Problems, all of which result in ethical issues and potential conflicts.   1. End of life decisions- Most of the physicians described conflict about withholding and withdrawing life-sustaining treatment. DNR decisions and futile treatment also emerged. 2. Communication and Hierarchical problems- Communication problems, hierarchical problems and paternalism emerged as ethical dilemmas 3. Social Problems- Economic barrier, inadequate staffing, priority of patients, social situation of patients, beliefs of physicians, limited resources, nursing home care, and care at home were reported   *Summaries only relevant to ICU Doctors | Physicians are more involved in managing scientific information related to disease while nurses focus on patients’ care and comfort, resulting in different priority list of ethical problems  Treatments that merely prolong dying and cause suffering were a source of great distress for these Physicians  The higher frequency of Physicians citing issues about communication with patient’s family and relatives compared with the nurses may indicate that physicians are more concerned with scientific processes than with patients’ relatives. However, it could also be patients and families feel more comfortable when discussing their concerns with nurses rather than with physicians  Physicians are encouraged to be decisive and to act with authority. Studies indicate that physicians view themselves as omnipotent  In this study, physicians did not follow a systematic pattern of ethical decision making. In solving ethical problems, physicians reported that they would ask colleagues one level up the professional hierarchy. Decisions were made in a narrow, habitual manner, through the elimination of the most significant and demanding elements of the decision making process, such as problem perception, information processing, gathering medical information, listing the alternatives and the possible consequences. | NA | 12 |
| S. Cohen et al., 2005 | Communication of end-of-life decisions in European intensive care units | Although cultural backgrounds may have an impact on these decisions, there are no studies of EoL communication and decision making involving and comparing a substantial number of European countries  OBJECTIVE: To examine end-of-life (EoL) practices in European ICUs: who makes these decisions, how they are made, communication of these decisions and questions on communication between the physicians, nurses, patients and families. | The main findings in this study are that the majority of ICU patients who are dying or are the subject of EoL decision making in European ICUs lack personal decision- making capacity, and that patient wishes in this respect are known in 20% of cases, usually from the family. There are also significant variations across Europe in practices regarding communication relating to EoL decisions  Subthemes:   1. it is not known whether the family’s knowledge of the patient’s wishes is truly that of the patient’s. Difficult to respect patient autonomy 2. In almost one-third of instances the EoL decision was made by a physician and was not discussed with the family 3. ICU physicians initiated discussion of EoL decisions in most of the instances 4. However, when discussions took place, the clinicians more often told the patients and families about the decision. 5. Agreement on the decision amongst physicians and the involved stakeholders in majority of cases | Public may take reassurance from data showing that ICU physicians have recognised the futility of life-sustaining treatments in patients, have been the first to initiate discussion about EoL decisions and have not supported persistence with a ‘mechanistic prolongation of death’    A paternalistic pattern of medical staff communication. More ‘telling’ compared to ‘asking’ in regard to EoL discussion with patient and family. Also, discussions did not occur with families in many cases because the patient was unresponsive to maximal therapy, or because the physician believed that the family would not understand.  However, telling families about EoL decisions when there is physiologically futile treatment is probably more appropriate than asking, as asking sends a mixed message, tends to confuse patients and families and undermines rather than enhances autonomy  Because of the legal uncertainty of some EoL practices in several European countries, the relative lack of communication may be secondary to physicians’ fear of charges of negligence or criminal actions | 9.5 | NA |
| A. Cottereau et. al  2016 | ICU physicians' and nurses' perceptions of terminal extubation and terminal weaning: a self-questionnaire study | Perceptions of death and dying by ICU nurses and physicians strongly influence EoL practices. These perceptions may differ depending on personal factors associated with a preference for either terminal weaning (TW) or terminal extubation (TE).  To the best of our knowledge, no published studies have been specifically designed to identify perceptions associated with preferences of ICU staff members for TW or TE.  The goal of this study of ICU staff was to identify perceptions associated with a preference for TW or TE after a decision to withdraw invasive mechanical ventilation (MV). | These findings were relevant to ICU physicians:   - 19.4 % of physicians had never or almost never performed TE and 5.0 % had never or almost never performed TW - 20.4 % felt there was a moral difference between withdrawing MV and withdrawing any other life-sustaining treatment - 28.0 % of physicians felt there was a moral difference between TW and TE - 14.3 % of physicians believed that MV withdrawal was mandatory after a decision to withdraw life support. - 7.6 % of physicians reported difficulties in discussing MV withdrawal with patients’ relatives   *Summaries only relevant to ICU doctors | Two previous studies suggested greater satisfaction and fewer complicated grief symptoms in relatives of patients who died after TE However, no studies specifically compared TW and TE in the ICU. Improved knowledge of the consequences of TW and TE would help to improve the quality of dying of ICU patients.  Physicians who preferred TE and those with no preference perceived TE as decreasing the medicalization of death and diminishing ambiguity. | 14 | NA |
| V. M. da Cruz, L. Camalionte and P. Caruso  2015 | Factors associated with futile end-of-life intensive care in a cancer hospital | Management of critically ill patients involves weighing potential benefit of advanced life support against preserving quality of life, avoidance of futile measures and rational use of resources.  Our study aims to identify the predisposing factors involved in the institution and maintenance of futile intensive care support in terminally ill cancer patients in whom no additional treatment for the malignant disease would be offered. | - Logistic regression analysis showed that the presence of a palliative care team was a significant protective factor for medical futility, while hematologic malignancy was a significant risk factor. - Family conference was not shown to be a significant protective factor - In 35 of the medically futile cases, futility could be traced to the attitude of the attending physicians, consisting in delayed or inexistent definitive directives, lack of adequate communication with families, ill-advised treatment.   *Summaries only relevant to ICU doctors | The results point to potentially avoidable situations that depend on a change in physicians’ attitudes.  Most cases of initiation of futile life support were traceable to the conduct of the attending physicians who failed to consider prognosis and allowed life support measures to be added in an automatic fashion as organic failures mounted. | 10 | 5 |
| B. J. Daly et. al  2018 | Complexity Analysis of Decision-Making in the Critically Ill | The ICU, viewed from the lens of complexity theory, is a complex adaptive system, dynamic and nonlinear, in which interactive processes (establishing goals of care and reaching decisions about the use of aggressive interventions and levels of treatment limitation) occur. These processes are sensitive to both initial or antecedent conditions and evolving behaviors. Thus, relationships and associations among variables change as new input is received and behavior adapts.  Our purpose of the study was to apply the principles of complexity science in examining how factors that influence decisions about transitions in care actually interact. | Results were analysed via 3 category of variables: - Antecedent characteristics of patients, family, and all physicians; process elements (expectations, valuation of treatment); and outcomes (focus of care and what was most important in making decision)   1. Antecedent characteristics: no physician characteristic was influential on expectations, evaluation of treatment, or focus of care at all 3 time points. Physician’s expectations for survival and cognitive status were the only consistent predictors of the focus at all time points. 2. Expectations/evaluation of treatment: expectation for survival was the only consistent element that influenced the evaluation of treatment effectiveness across time. 3. Outcomes: In terms of what was most important to the physician in making medical decisions, only expectations for future functional status and the patient’s Charlson score were consistent influences.   At no time did family variables show a significant relation to physicians’ expectations, what was important to the physician, nor the physicians’ evaluation of treatment effectiveness.  *Summaries only relevant to ICU doctors | Expectation for survival was the most important predictor of the focus of care.  The outcome that was identified by physicians as most important was not significantly related to focus of care.  This study provides important insights into the dynamic process of decision-making in the real world and demonstrates the persistent power of a culture that prioritizes length of life over quality of life. | 10.5 | NA |
| S. M. Donnelly and A. Psirides  2015 | Relatives' and staff's experience of patients dying in ICU | The quality of dying and death (QODD) survey  used in ICU studies has been criticised for its limitations including that family members rated the questionnaire 6/10 for difficulty and several items were not answered by a majority of family members. A different research approach is warranted. Our study is unique in conducting face-to-face interviews with families and ICU staff exploring their experience at this important time. | Main themes that emerged from the doctors’ interviews were:  Quotes are summarized in Table 4. according to their respective themes   1. Their burden of prognostic uncertainty - Four registrars describe struggling with decision making. In one case, with all ICU consultants and neurosurgeons agreeing on a very poor prognosis, one remains uncertain. 2. Limited time available to establish relationship with the patient or family 3. Their view on dignity 4. Personal impact on doctor - A senior ICU doctor reflects on a day when three patients died: ‘It’s certainly one of those days where you do get quite emotionally wiped out compared to other days’. (D6) 5. Remaining professionally detached or not - Three junior doctors stated that they do not get emotionally involved.   *Summaries only relevant to ICU doctors | Some doctors are clear as to their role, satisfied with how the patient’s death unfolded and believe that emotional involvement is unhelpful to their role. Other doctors struggle with the nature of decision making, with prognostication, reflect on what they could have done better, recognize the burden, express wish to be more involved as the patient is dying, speak of their own humanity and describe humanity of family scene. | NAA | 17 |
| N. C. Ernecoff, F. A. Curlin, P. Buddadhumaruk and D. B. White  2015 | Health Care Professionals' Responses to Religious or Spiritual Statements by Surrogate Decision Makers During Goals-of-Care Discussions | Although many patients and their families view religion or spirituality as an important consideration near the end of life, little is known about the extent to which religious or spiritual considerations arise during goals-of-care conversations in the intensive care unit.  The objective of this study is to determine how frequently surrogate decision makers and health care professionals discuss religious or spiritual considerations during family meetings in the intensive care unit and to characterize how health care professionals respond to such statements by surrogates. | 4 themes frequently arose in response to religious or spiritual statements by surrogates:   1. Health care professionals most commonly responded to surrogates’ religious or spiritual statements by speaking about the medical plan for treatment or goals of care, including terminal- event planning and implications for care. 2. Health care professionals responded with empathic statements. physician responses rarely directly addressed surrogates’ spiritual or religious language. 3. Acknowledging surrogates’ religious or spiritual statements with closed-ended responses, such as, “Mhmm,” or, “Okay”. 4. Few health care professionals responded to religious statements by emphasizing their commitment to high-quality medical treatment, reassuring surrogates regarding their own dedication to the patient with a statement containing more emotional support 5. Less frequent themes also emerged: responding by exploring patient’s or surrogates’ personal spirituality, voicing his own religious beliefs by agreeing.   Responding to a hope for miracle by changing the subject, expressing empathy, or closed ended statements.  Content of Health Care Professionals’ Religious or Spiritual Statements:   1. Most common – offering sources of religious or spiritual support 2. Asking about the importance of religion to patient or surrogate 3. Occasionally voicing their own religious or spiritual beliefs to surrogates   *Summaries only relevant to ICU doctors | Discussion of religious or spiritual considerations occurred in fewer than 20% of goals-of-care conferences and health care professionals rarely attempted to explore the patient’s or family’s religious or spiritual ideas.  Health care professionals may avoid these topics because they feel unprepared to engage patients and surrogates.  Previous studies found that a central barrier to discussing such concerns is a lack of training for discussing religion and spirituality. Another possibility is that health care professionals may place less value on such discussion either because of their own attitudes toward religion and spirituality or because they perceive the decisions as depending solely on medical facts in- stead of including diverse values. | NA | 14 |
| E. Ferrand et al., 2003 | Discrepancies between perceptions by physicians and nursing staff of intensive care unit end-of-life decisions | Several studies have pointed out ethical shortcomings in the decision-making process for withholding or withdrawing life-supporting treatments. We conducted a study to evaluate the perceptions of all caregivers involved in this process in the intensive care unit. | 1. ICU Commitment to High Ethical Standards- Physicians believed that their ICU was committed to high ethical standards. 2. Satisfaction with the Process for Making Decisions to Forego Life-Sustaining Treatment- DFLST processes were believed to be always satisfactory by majority of physicians. Satisfaction was significantly associated with perceptions of a commitment of the ICU to high ethical standards, regular meetings to discuss ethical issues and presence of a psychologist in the ICU staff 3. Decision-Making- Majority stated decision should be collaborative but reported that occurrence rates is lower in actual practice. 4. Communication with the Family- Only 61% of physicians believed that families should be informed fully, the main reason cited is that it might add to the family’s distress 5. Criteria for Decisions to Forego Life-Sustaining Treatments- Futility and no hope for future quality of life care were the reasons most often cited by physicians 6. Liability- Physicians believed that nursing staff in charge of the patient should share legal responsibility. Although most physicians did not believe that they were breaking the law when implementing DFLSTs, some were still worried of malpractice suits.   *Summaries only relevant to ICU Doctors | Physicians were more likely to be satisfied with decision making process in medical ICUs than in Medical-surgical or surgical (lowest)  Fear of litigation clearly had an unfavorable influence on DFLST procedures.  Concern about litigation was one of the reasons given by physicians for modifying the information they provided to competent patients. Inaccurate information was also given to nurses by physicians  Decisions made openly and discussed in depth withal those involved may be less likely to lead to litigation, rather the opposite. | 8 | NA |
| E. Festic, M. E. Wilson, O. Gajic, G. D. Divertie and J. T. Rabatin  2012 | Perspectives of physicians and nurses regarding end-of-life care in the intensive care unit | The delivery of end-of-life care (EoLC) in the intensive care unit (ICU) varies widely among medical care providers. The differing opinions of nurses and physicians regarding EoLC may help identify areas of improvement.  The objective of this study is to explore the differences of physicians and nurses on EoLC in the ICU and how these differences vary according to self-reported proficiency level and primary work unit. | The results suggested differences between staff physicians and fellows in the following ways:   1. Fellows were more likely to agree than attending physicians that “Fear of litigation often results in transferring dying patients to ICU” 2. Fellows were more likely to agree than attending physicians that “The ICU provides professional education to physicians to improve end-of-life clinical practices”   *Summaries only relevant to ICU doctors | Attitudes of physicians regarding EoLC evolve with training and experience. This may reflect the fact that one of the best methods of learning to provide quality EoLC is through experience.  Almost no physicians thought that a patient’s death in the ICU reflects a medical failure. Most physicians thought that caring for dying patients and their families was a rewarding experience. This was present despite the above listed sources of dissatisfaction. Agreement may be secondary to recent institutional efforts to provide comprehensive EoLC training to physicians. | 6.5 | NA |
| R. Fumis, G. De Paula Pinto Schettino and T. Domingos Corrêa  2018 | Would you like to be admitted to the ICU? Intensivists' and the general public's preferences according to different outcomes | Discussions about invasiveness of care (advanced directives) and end-of-life issues have become frequent among intensivists and patients. Nevertheless, there are considerable divergences in the attitudes between intensivists and patients toward end-of-life care in the intensive care units (ICU).  This study aims to compare the preferences between intensivists (ICU physicians) and general public regarding ICU admission of a hypothetical critically ill patient with six different clinical outcomes, ranging from ICU discharge without any neurological sequelae, nor dependence for daily activities, to death. | 163 intensivists were asked to decide on ICU admittance for 6 hypothetical scenarios. 1 group of intensivists was to choose for themselves and 1 group for their patients.   1. In all scenarios, except when the outcome was severe disability (Scenario 5) or death (Scenario 6), intensivists were more likely to propose ICU admission for their patients rather than for themselves 2. The intensivists' opinion choosing for their patients did not differ from the general public's opinion when the clinical outcome was death (Scenario 6)   *Summaries only relevant to ICU doctors | We found that intensivists often would not make the same choices for their patients they would make for themselves. It is possible that intensivists choosing for themselves are more prone than the general public to take risks when the expected out- come is only mildly disabling but less prone to take risks when the out- come is severe disability. In contrast, general public has a more uniform approach.  Considerable divergences between intensivists' and patients' preferences toward end-of-life care exist. Advanced care planning and effective ongoing communication among intensivists, patients and relatives are essential to improve end-of-life decisions and the quality of care. | 13 | NA |
| B. Gatta and J. Turnbull  2018 | Providing Palliative Care in the Medical ICU: A Qualitative Study of MICU Physicians' Beliefs and Practices | With the current and projected shortage of palliative care (PC) specialists, an integrative model of PC will be needed to meet the needs of patients in intensive care units (ICUs). Prior studies of PC interventions suggest that success depends upon meeting the needs of individual institutions or ICUs.  The objective of this study was to qualitatively explore the beliefs and practices of one institution’s medical ICU (MICU) physicians in regard to providing an integrative model of PC. | Three major themes were identified:   1. Embracing of primary PC by the critical care physician – all affirmed that PC was an important part of practice and most (9 out of 17) expressed that providing PC gave them professional or personal satisfaction. 2. Added value of the PC consultant - They were not only able to name the stated core services of the PC consultant team but recognized that the PC team could provide benefits such as “holistic care”, patient and family education, and longitudinal patient care. 3. Motivations for moving from primary PC to initiating PC consultation - The most frequently acknowledged reason was lack of time on the part of the MICU physician. Another major motivation was MICU team’s need for a second opinion or the fact that a team member or family member had proposed a PC consult. | They hypothesized that in this population, MICU physicians may appreciate further educational or institutional support to improve their own primary PC skills (since they see it as part of their practice and find meaning in it) and that further programming to promote use of the PC team may not make a large impact (because the PC consult team is already seen as a key consulting service) | NA | 24 |
| K. M. Gutierrez  2012 | Advance directives in an intensive care unit: experiences and recommendations of critical care nurses and physicians | The ICU is a setting where the effectiveness or lack of effectiveness of advance directives (ADs) is clearly seen and where the need for advance care planning is critical, a descriptive study focusing on ADs in an ICU, obtained by ethnographic means, was implemented to illuminate this topic.  This study explored the experiences of critical care nurses and physicians with advance directives (ADs) in an intensive care unit (ICU) to identify the benefits and limitations of ADs and recommendations for improvement. | These themes relevant to the limitations of ADs emerged:   1. Prevention of aggressive treatments - One significant problem described by physicians is that ADs often do not prevent unwanted aggressive treatments. 2. Addressing clinical situations - ADs usually do not address the specific issues that a majority of patients face in real life 3. Vague and confusing terminology - The terminology used in most ADs was described as confusing and lacking in a level of specificity that is necessary to facilitate decision making. 4. Physician identification of a terminal diagnosis or prognosis - Most of the ADs found in the patients’ medical records in the ICU included the prerequisite of a terminal diagnosis or prognosis first before the wishes described in the living will would be considered. 5. Family member difficulty “letting go” and physician legal concerns - Many families struggled with being able to “let go” of their loved one and allow him or her to die. This has led to families demanding that physicians implement aggressive treatments that were not consistent with patient wishes in ADs and/or their code status. Physicians expressed concerns regarding potential legal action imposed by families if they refused to comply with family demands. Thus, they described “caving” in to families, even when their actions clearly and directly conflicted with patients’ verbal and/or writ- ten wishes. 6. Patient fear of sharing AD – Patient was afraid that the physicians would “throw in the towel too early” 7. Discomfort with discussing EoL - Providers brought up the fact that many patients and families are reluctant to talk about EoL.   These themes relevant to the strength of ADs and provider recommendations emerged:   1. Patient discussions with family - Providers perceived that advance care planning should be about individualized discussions between patients, families, and providers and should be prioritised over ADs 2. Initiation of EoL and QOL discussions - Providers believed that discussions of EoL and QOL should be initiated and encouraged by patients’ primary care providers during routine clinic visits before patients experience a health crisis. 3. Shifting responsibility for decision making and outcomes – ADs served to shift the moral burden for decision making and outcomes of these decisions off of families and providers and place it on the patient.   *Summaries only relevant to ICU doctors | An unexpected impact of ADs was a patient’s fear of physicians “throwing in the towel” too early which can be seen to be a concern of ADs actually decreasing patient autonomy. This brings to the forefront the primary im- portance of trust and communication in the patient-physician relationship in relation to limitation of life-sustaining treatments.  Physicians perceived the use of ADs as significantly reducing stress and guilt for both families and providers and facilitating EoL decision making  Suggested implementations:   - Develop a decision tree to assist physicians and head off conflicts - Lobby for legislation that promotes financial reimbursement to primary care providers for discussing EoL and QOL as part of advance care planning. - Lobby state legislatures to adopt or adapt systems to facilitate communication and recognition of physician orders for advance care planning from one setting to another. | NA | 17 |
| A. B. Hamric and L. J. Blackhall, 2007 | Nurse-physician perspectives on the care of dying patients in intensive care units: collaboration, moral distress, and ethical climate | OBJECTIVE: To explore registered nurses' and attending physicians' perspectives on caring for dying patients in intensive care units (ICUs), with particular attention to the relationships among moral distress, ethical climate, physician/nurse collaboration, and satisfaction with quality of care. | Several themes were examined on physicians’ perspectives   1. Moral Distress- Physicians did experience some moral distress when attending to dying patients. The most distressing situations for both groups involved caregivers feeling pressured to continue aggressive treatment in situations where they did not think such treatment was warranted. Working and assisting incompetent physicians also causes moral distress. One doctor had considered leaving a clinical position due to moral distress 2. End-of-Life Communication- Majority of physicians have been urged by nurses to get a patient’s family to agree to a DNR so futile therapy can be withdrawn. Withholding diagnostic/prognostic information, nurse frustration with physician communication, and satisfaction with patient/ physician communication were areas of disagreement between RNs and MD 3. Ethical Environment, collaboration and satisfaction of care- MDs rated the both as better than did their RN colleagues. 4. Correlations Among Variables- collaboration was correlated with satisfaction with quality of care and ethical environment for doctors   *Summaries only relevant to ICU Doctors | Majority of the items listed as the highest sources of moral distress by both MDs had to do with pressure to continue aggressive treatment in situations where they did not think such treatment was warranted. This represents a change from the beginnings of the bioethics movement, when ethical conflicts (including the cases of Karen Ann Quinlan, Paul Brophy, and Claire Conroy) generally involved situations where the medical team insisted on aggressive care despite patient or family requests to withdraw it.  The attending MD, however, must sign the order to withdraw life support and thus feels the burden of responsibility for such decisions and for the possibility that withdrawal of care may prematurely end the life of a patient who could have been saved  Experiences with patients who unexpectedly improved in response to aggressive therapy are powerful examples used by MDs to justify aggressive therapy in difficult cases  MDs are more concerned with the survival of the few  MDs tend to believe that they are collaborating well, even when their nursing colleagues disagree, general exhortations to “collaborate” will not improve the interactions between RNs and MDs around EoLC | 11 | NA |
| L. A. Hawryluck, W. R. Harvey, L. Lemieux-Charles and P. A. Singer, 2002 | Consensus guidelines on analgesia and sedation in dying intensive care unit patients | BACKGROUND: Intensivists must provide enough analgesia and sedation to ensure dying patients receive good palliative care. However, if it is perceived that too much is given, they risk prosecution for committing euthanasia. The goal of this study is to develop consensus guidelines on analgesia and sedation in dying intensive care unit patients that help distinguish palliative care from euthanasia. | In the third round, consensus was obtained in both Delphi (Intensivist and coroner) panels on 16 statements. These statements can be organized into 4 categories.  The first category discusses:   1. How palliative care is different in the ICU than in other settings- The palliation of dying patients in the ICU is different from palliative care in other settings since the dying process tends to be more dramatic and the time from withholding/withdrawing active disease treatment to death is much shorter 2. The role of palliative care in the ICU- The goals of palliative care in the ICU are: 1) relief of pain, 2) relief of agitation and anxiety, 3) relief of dyspnea, 4) psychological and spiritual support of patient and family and, 5) provision of comfort 3. The difficulties in assessing pain and suffering that are specific to the ICU- Pain and suffering assessment is difficult yet crucial, especially in the ICU setting. Poorly taught skills in assessing pain. Education, research and discussions with family members may help physicians improve.   The second category of consensus statements describes the management of pain and suffering in the ICU   1. Relief of Pain and Suffering- Both pharmacological and non-pharmacological means should be used. Presence of family, friends and pastoral care as well as accommodating patients' religious and cultural beliefs also play an important role in alleviating pain and suffering 2. Drugs- The amount of drugs needed varies on an individual basis, treatment MUST be individualized. The total amount of drugs required for any individual patient may far exceed any preconceived notions of usual doses. The goal of palliative care is to provide relief of pain and suffering and whatever the amount of drugs that accomplishes this goal is the amount that is needed for that individual patient. When appropriate doses of narcotics and sedatives are used and the intent of the physician is clear and well documented, pre-emptive dosing in anticipation of pain and suffering is not euthanasia nor assisted suicide but good palliative care.   The third category of consensus statements focuses on areas of controversy in the current literature.   1. Special situations- Neuromuscular blockers mask the clinical signs of pain and suffering, withholding and withdrawal of life support should be started after effects wear off to permit Intensivists to accurately assess pain and suffering and ensure good palliative care. They should not be started in order to hide patient distress. Patients in persistent vegetative states are deemed incapable of feeling pain or anxiety, sedatives and narcotics are usually not required during the withholding/withdrawal of life support. The family's perceptions of pain and suffering may play a role in the use of narcotics and sedatives in these patients. 2. Terminal Sedation- Since terminal sedation may arguably make the detection of euthanasia/ assisted suicide more difficult, the intent of the Intensivist is crucial 3. Intent- The intention of the Intensivist administering narcotics/sedatives to palliate dying patients can be assessed by careful documentation. Intensive care units should develop guidelines governing the process of withholding and withdrawal of life support and Intensivists should justify and document any need to deviate from the policy and the anticipated modifications 4. Principle of double effect- If the amount of narcotics/sedatives required to relieve pain and suffering at the end-of-life may foreseeably cause hastening of death, although the physician's intent is solely to relieve suffering, this should be considered palliative care. 5. Distinction between Palliative Care and Euthanasia- The intent of the physician administering narcotics and sedatives to the dying patient is the most crucial distinction   The fourth category of consensus statements discusses how palliative care in the ICU can be improved in the future through research and ongoing education of the staff   1. Better pain assessment and treatment- Open discussions involving all members of the health care team and family, consulting and sharing when faced with difficult cases, improvements in education and research are needed. The development of a process to review our performance in palliative care within each ICU and national consensus guidelines will also improve our skill in assessing pain and suffering and improve our abilities to relieve it at the end-of-life. 2. Support for Intensive Care staff- psychological and emotional support for the ICU staff by Developing a supportive working group, open communication and regular debriefings. The ICU social worker, pastoral care representative and, within the hospital, the departments of psychiatry and psychology may also be helpful 3. Palliative Care Medicine Consultation | Healthcare providers withdrawing treatments from dying patients frequently encounter apprehension and concern from surviving family members about how their loved one's pain and distress will be treated  Questions by family members often reveal concern that their loved one's final moments will be filled with suffering, or, alternatively, that drugs such as opiates and benzodiazepines given by the healthcare team will unduly hasten the dying process and/or kill their loved one  Our consensus guidelines on analgesia and sedation in dying ICU patients are the first of their kind developed using consensus methods and involving coroners. The use of consensus methodology and the participation of coroners will hopefully serve to clarify the areas of controversy, their nature and help us resolve these dilemmas in the future | NA | 9 |
| N. J. Henrich et al., 2016 | Causes of moral distress in the intensive care unit: A qualitative study | Although moral distress can be evaluated by both quantitative and qualitative means, there have been few qualitative studies of moral distress in ICU professionals and none that have addressed moral distress in all ICU professionals in both community and tertiary ICUs. The purpose of the study is to examine the causes of moral distress in diverse members of the intensive care unit (ICU) team in both community and tertiary ICUs. | The common causes of moral distress fell into 5 main categories for physicians:  Quality of care   1. Concerns about other providers’ care 2. Teaching vs. optimal care- Physicians expressed concern that patients may not receive the best or most appropriate care when care was provided by residents 3. Lack of end of life conversations   Amount of care provided   1. Too much care provided--physician’s choice- Physicians find it hard when they have someone who they think they are doing too much to, but Not know for sure 2. Too much care provided--family’s choice 3. Too little care provided--physician’s choice- Felt care was withdrawn from a patient inappropriately because the team had become pessimistic about patients‟ likelihood of survival.   Poor communication  Interactions and conflict with families  Lack of support and resources   1. Lack of resources - Physicians felt conflicted about trying to provide best patient care while also being concerned about the limited resources 2. Lack of support from management   *Summaries only relevant to ICU Doctors | Physicians were morally distressed by patients being denied the information that they were dying and the lack of discussion about end-of-life care. ICU physicians were often left to break this news to the patients  Interventions to address moral distress should consider these specific causes, and the target groups and settings where they apply.  Physicians described feeling distressed when patients survived who had not been expected to recover because it made them wonder if there were other patients who may have survived if care had been continued or more aggressive | NA | 17 |
| C. L. Hough, L. D. Hudson, A. Salud, T. Lahey and J. R. Curtis, 2005 | Death rounds: end-of-life discussions among medical residents in the intensive care unit | PURPOSE: We introduced "Death Rounds," a monthly discussion of the issues and emotions surrounding the care of dying patients, into the intensive care unit (ICU) rotations for medical house staff. We surveyed participating residents to evaluate their satisfaction with these discussions. | Summary of survey results:   1. Majority agreed Death Rounds were worthwhile compared with other teaching sessions or conferences 2. Majority agreed or are neutral about Death Rounds improving ability to take care of dying patients 3. Majority felt their ability to cope with dying patients has improved because of Death Rounds 4. Majority agreed Death rounds has improved ICU experience and should be incorporated into ICU rotations for residents 5. Majority disagreed that too much time was spent discussing end of life issues | Residents were free to ask basic questions about dealing with patient death that were not typically addressed on morning rounds  Residents were also encouraged to delve into ethical and emotional issues; this evolution seemed to occur naturally at each session. Even the quieter residents were able to express feelings and concerns in these sessions; difficult revelations were met with empathy and support.  There were several themes that recurred with frequency in Death Rounds discussions. These themes included dealing with deaths of young patients, residents’ concerns that they had not done everything possible to prevent the death of a patient, the frustration of working with families with unrealistic expectations, and the difficulty of guiding families in end-of-life decision making in the setting of medical uncertainty | 8 | NA |
| H. I. Jensen, J. Ammentorp, H. Johannessen and H. Ørding, 2013 | Challenges in end-of-life decisions in the intensive care unit: an ethical perspective | When making end-of-life decisions in intensive care units (ICUs), different staff groups have different roles in the decision-making process and may not always assess the situation in the same way. The aim of this study was to examine the challenges Danish nurses, intensivists, and primary physicians experience with end-of-life decisions in ICUs and how these challenges affect the decision-making process. | Two main categories of challenges were identified “collaboration” and “future perspectives for patients.”:  Collaboration   1. Treatment Potential- Difficulty assessing whether the patient was a candidate for intensive therapy or when care is futile 2. Changes and Postponements of Withholding and Withdrawing Therapy Orders- Lack of continuity among the staff was sometimes a reason for end-of-life decisions being changed or postponed 3. Decision-Making Process- The need for interdisciplinary collaboration was stressed by all   Future Perspectives for Patients   1. Patient’s Wishes- different opinions were identified concerning the right time to discuss EoL issues with the patient. Difficulty of assessing whether the patient was competent to make a decision 2. Outcome- For patients unable to voice opinions, physicians find it challenging to make decisions on their behalf and assess what would be in the patient’s best interest 3. Treatment suffering- how long should treat patients, knowing that many will not survive and are suffering   *Summaries only relevant to ICU Doctors | Intensivists felt that the primary physicians wanted them to make decisions they did not want to make themselves and that the parent wards did not often enough discuss patients’ wishes regarding the level of therapy with the patients. The ICU staff found that the parent ward should more often initiate these talks before an acute situation emerged  Only one or two decades ago many patients died of conditions that can be cured today. If these patients had been left to die, knowledge about new therapy options would not have been obtained. However, the price for obtaining the knowledge has, to a certain extent, been paid by patients undergoing prolonged ICU therapy only to ultimately die of their illness  Interviews suggest that both admittance to the ICU and continuation of futile intensive therapy may sometimes be due to lack of will and ability to discuss end-of-life issues with the patients and lack of medical assessment of the patient’s possibility to benefit from intensive therapy. | NA | 17 |
| G. M. Joynt, et al., 2015 | The Durban World Congress Ethics Round Table IV: Health care professional end-of-life decision making | Introduction: When terminal illness exists, it is common clinical practice worldwide to withhold (WH) or withdraw (WD) life-sustaining treatments. Systematic documentation of professional opinion and perceived practice similarities and differences may allow recommendations to be developed. | These are the statements of general agreement   1. Knowledge of local, cultural, and religious practice and expectations as well as local legal restrictions should inform individual practice. 2. Goal-of-care discussions should generally occur early (within 48 h) after the patient's admission to ICU. Care should be taken to specifically address WH/WD in appropriate circumstances. Special sensitivity should be exercised when interviewing certain patients/surrogates of certain cultures 3. Physicians are the most likely to initiate discussions that may lead to WH/WD decision-making processes. The wishes of patients themselves and families have the right to trigger WH/WD discussions. 4. When possible, treating ICU physicians and other specialty medical physicians should reach consensus that a WH/WD discussion is reasonable before formally starting a discussion with the patient/surrogate. In most parts of the world, nurses and other health care staff who are meaningfully involved in the patient's long-term care should also be consulted. 5. Consensus regarding the decision to trigger a WH/WD discussion is usually reached by discussion between relevant health caregivers on routine daily ward rounds. Additional formal discussion forums can be scheduled if necessary. 6. The decision to trigger a discussion of WH/WD LST should be made based on the principle that the patient's best interest is no longer served by LST, that occurs when sufficient net medical benefit cannot be achieved by ongoing LST, such as quality of life issues 7. The following may serve as triggers for initiating WH/WD discussions with the patient/surrogate. A WH/WD discussion: Expected time of survival is short, multiple organ failure, poor long term outcome 8. Neither age alone, nor severe illness should be considered a sufficient trigger to initiate WH/WD discussions.   *Summaries only relevant to ICU Doctors | Legal challenge, a known concern for ICU clinicians in certain Asian countries  Asian countries, where discussion of death may be considered rude or even dangerous by inducing future “bad luck” and undermine the filial duty of children to protect elders and parents  Comments accompanying the disagreeing responses stressed that subsequent decisions to take action should always follow discussions with surrogates and the wider health care team and not only result from the opinions of the treating physician, whose religious or cultural values might be different than the patient's.  Although advance directives are legally regulated in some countries, they are generally respected but not legally regulated in others; and, thus, levels of implementation may vary  Members from Middle Eastern and Asian regions expressed the need to be aware of local, religious, and legal constraints when considering WH/WD discussions  Recently, however, as part of a system of “triage” or prioritization during infectious outbreaks, it has been suggested that patients not responding positively to ICU care and with resultant poor prognosis should have life support withdrawn by discharge from the ICU. Under these extreme circumstances of resource stress, it appears that prioritization of resources to maximize overall resource use at end of life may be considered acceptable | NA | 10 |
| A. Laurent, M. Bonnet, G. Capellier, P. Aslanian and P. Hebert, 2017 | Emotional Impact of End-of-Life Decisions on Professional Relationships in the ICU: An Obstacle to Collegiality? | OBJECTIVES: End-of-life decisions are not only common in the ICU but also frequently elicit strong feelings among health professionals. Even though we seek to develop more collegial interprofessional approaches to care and health decision-making, there are many barriers to successfully managing complex decisions. The aim of this study is to better understand how emotions influence the end-of-life decision-making process among professionals working in ICU. | Three themes emerged from the interviews with Physicians  Experience of the end-of-life decision-making process   1. Sense of responsibility in decision-making 2. Difficulty in making end-of-life decisions   Impact of the experience on care relationships   1. Family as a decision-making partner 2. Identifying with the family - Conflicting views on continuing aggressive treatment poses an ethical challenge. Doctors can spend hours meeting families, every day to attempt, not to convince them but to make them understand their point of view, tiring them out. 3. Suspending the decision according to family expectations 4. Lack of understanding of family expectations   Impact of the experience on professional relationships   1. Decision-making under pressure 2. Needing to reach a consensus with nurses   *Summaries only relevant to ICU Doctors | The absence of objective and validated criteria leads to uncertainty and seems to compel the physician to devote him/herself to the family to compensate for the lack of data but the family introduces strong emotional dimensions into the care decision which hinder collegial decision-making.  Two conflicting ideals emerge: avoid making the family suffers and avoid making the patient suffers. These emotions interfere with nurse-physician interactions and maintain persistent intrapersonal and interpersonal conflict among professionals.  End-of-life decisions involve emotional proximity between patients/families and health professionals. These emotional dimensions, however, must be shared as a team and validated as a “professional stance.” | NA | 18 |
| N. S. McAndrew and J. S. Leske 2015 | A Balancing Act: Experiences of Nurses and Physicians When Making End-of-Life Decisions in Intensive Care Units | Uncertainty in the ICU over whether to provide or withhold advanced medical support is one of the most common sources of ethical conflicts in the workplace, as identified by ICU nurses and physicians.  The purpose of this qualitative, descriptive study was to describe end- of-life decision-making experiences as understood by critical care nurses and physicians in intensive care units (ICUs). | The overarching theme found was end-of-life decision making is a balancing act.  Three subthemes emerged:   1. Emotional responsiveness – The role emotions can play in both relating to patients and causing conflict with patients and their family. 2. Professional Role and Responsibilities – Being available and helping family members move forward with a decision 3. Intentional Communication and Collaboration – Communication between nurses and physicians with the goal of shared decision making is important in avoiding frustration | A lack of collaboration in shared decision making can lead to conflict between nurses and physicians.  Distress can in physicians occur when families have expectations incongruent with patient prognosis.  Physicians need support with EoL decision making. Interdisciplinary collaboration has been shown to improve patient care. | NA | 15 |
| H. M. Mehter, J. A. Clark and R. S. Wiener 2015 | Physician approaches to provider-family conflict associated with end-of-life decision-making in the ICU: A qualitative study | Disagreements between physicians and families over end-of-life care are common and can lead to significant stress for all parties. Conflict has been independently associated with both provider burnout and family post- traumatic stress disorder.  Hence, we sought to elucidate the ways in which ICU physicians approach communication and decision-making when there is conflict with families, as well as the emotional impact on families and physicians themselves. | Physicians described several strategies to minimize or avoid conflict:   1. Preparing for the Family Meeting: “Sizing Up” the Family – Preliminary evaluation of the family’s state of mind and priorities 2. Initial Approaches during Formal Family Meetings    1. Completing the narrative    2. Offering an opinion 3. Managing Disagreement    1. 1:Acquiescing to families    2. 2: Persuading families   Modulating Factors-  Factors physicians identified that impacted the aggressiveness of their persuasion:   1. Mismatch between family and patient preferences 2. Patient discomfort 3. Physician’s experience 4. Families’ emotional well-being   Physicians’ Emotional Responses:   1. Positive emotions with clarity and resolution 2. Anxiety and emotionally draining when anticipating conflict | Our study suggests that although physicians do take family preferences into account, their approach  to decision-making may be driven by the goal of achieving the outcome that the healthcare team believes to be in the patient’s best interests.  Despite their best efforts and intentions, at times physicians engaged in behaviours that were potentially unhelpful or even harmful to families.  Easily implementable interventions such as “death rounds” for periodic debriefing and reflection may help ease ICU clinicians’ emotional burden.  Recent work has begun to quantify key aspects of palliative care specialists’ approach to managing conflict that may help explain the benefit of palliative care involvement and that could be the focus of interventions to improve critical care clinicians’ conflict resolution skills  Our findings also underscore the need to support clinicians engaged in emotionally draining encounters. | NA | 19 |
| M. C. Monteiro, A. S. Magalhaes, T. Feres-Carneiro and R. N. Machado  2016 | Terminality in the ICU: The emotional and ethical dimensions of the medical care of the intensivist | Doctors anguish themselves for having to save the life of the patient at all costs, for having to take decisions about refusal or suspension of the treatment and, frequently, they feel alone, powerless and with difficulties in addressing the family members, who ask constant questions about the evolution of the patient.  The purpose of this article is to understand the repercussions about the terminality of the patient in ICU among the medical team, focusing on the emotional and ethical dimensions. | 1. Perception about the patient in situation of terminality – Attitudes towards treatments are based on perceived prognosis 2. Emotions in the face of death and dying – Physicians emotionally detach from their patients to minimize personal impact   Ethical conflicts – medicolegal concerns and differing family values are sources of ethical conflicts and dysthanasia | It is highlighted the importance to create spaces to listen especially in the ICUs– institutional spaces that address the pain of the “wounded healer”.  Based on the results of this research, it is also suggested the expansion of the discussion on death and dying in the society, including ethical and bioethical aspects – euthanasia, dysthanasia, orthothanasia, living will –, and the dissemination of the palliative care and its philosophy that contemplates the ill patient in his totality.  A solution is the use of use of Advance Directives. However, a cultural and institutional change is necessary, because we still have the physician as the main decision maker | NA | 15 |
| L. Nordgren and H. Olsson, 2004 | Palliative care in a coronary care unit: a qualitative study of physicians' and nurses' perceptions | BACKGROUND: Earlier research has shown that physicians and nurses are motivated to provide good palliative care, but several factors prevail that prevent the best care for dying patients. To provide good palliative care it is vital that the relationship between nurses and physicians is one based on trust, respect and sound communication. However, in settings such as a coronary care unit, disagreement sometimes occurs between different professional groups regarding care of dying patients. AIM AND OBJECTIVES: The aim of this study was to describe and understand physicians' and nurses' perceptions on their working relationship with one another and on palliative care in a coronary care unit setting. | 3 main concepts was identified from the data analysis  A dignified death   1. Integrity- By respecting the patients’ integrity, the patients’ human dignity could be preserved 2. Participation in care- patients’ and the relatives’ participation in care as one way of expressing respect for the patients’ integrity 3. Increasing patients’ well-being- If the patients and their relatives felt safe, comfortable and cared for, this would help to increase their well-being 4. Alleviating suffering   Prerequisites for providing good quality palliative care   1. Teamwork- Physicians with SNs and RNs 2. Work experience and self-reflection- Physicians believed that professional experiences made them more qualified in assessing the patients’ condition and in providing efficacious care to patient   Obstacles to providing good quality palliative care   1. Inappropriate ward environment- Informants considered problems associated with the ward environment and the unit’s organization as serious obstacles to providing good palliative care 2. Lack of clear guidelines on palliative care- Commonly used concepts and terms in documentation were ‘do-not-resuscitate’ or ‘palliative care only’. Such terms can have different significance for different groups or persons. 3. Insufficient knowledge on palliative care 4. Difficult dialogues- communicating with people with severe, incurable disease or with people in shock or grief is exceedingly difficult, and they may not register the information told to them 5. Unnecessary suffering- The informants sometimes had to perform procedures that were painful yet unnecessary 6. Disagreement between different professional groups- Conflict between nurses and physicians   *Summaries only relevant to ICU Doctors | Work experience and self-reflection were viewed as processes that allow Physicians to feel comfortable with implications of death or dying.  Results illuminate the fact that there is a strong need for support and education concerning end-of-life-care  Deficient guidelines on the palliative care of dying patients, together with the confusion of the concepts of palliative care and life-sustaining treatments, resulted in disagreement and conflicts between caregivers  One strategy that would benefit caregivers is if time and space are assigned in the workplace for professional conversation and reflection over care and over the relationships among different categories of professionals. | NA | 13 |
| S. Y. Park, J. Phua, M. Nishimura, Y. Deng, Y. Kang, K. Tada, Y. Koh  2018 | End-of-Life Care in ICUs in East Asia: A Comparison Among China, Korea, and Japan | Ethical considerations in EoL care in China, Korea, and Japan have roots in Confucian thought, resulting in family-based and harmony-oriented ethical systems. In spite of their cultural similarities, however, China, Korea, and Japan have different political, economic, and healthcare systems, in addition to levels of Westernization. As data on this topic are comparatively sparse, it is unclear how the combination of these factors influences EoL care in these countries. In the current analysis, we aimed to compare physicians’ perceptions and practice of EoL care in ICUs in China, Korea, and Japan.  We hypothesized that while there would be similar social cultures anchored on Confucianism, other factors would result in differences in EoL care between the countries.  As our ultimate goal was to prepare a regional guideline on EoL care, it was important to under- stand these factors. | 1. Differences in Withholding and Withdrawing LST Between Countries – Country was an independent predictor in the use of DNRs, and differences were seen in TPN, antibiotics, dialysis and suctioning. Country was not significant in predicting the use of mechanical ventilators 2. Factors to Consider When Withholding and Withdrawing LST – Patients’ wishes, financial burden to institution, attitudes towards legislation, legal risks 3. Differences in Responses To Case Scenarios Between Countries 4. Differences in EoL Care Between the Respondents – Age, gender, clinical experience, pulmonology specialty and religious backgrounds were significant predictors for EoL decisions | Although there were a few exceptions, we noted a trend, in which responses from Korea tended to be between those from Japan and China.  Westernization in each country may account for the differences in attitudes towards EoL care, alongside healthcare system, economic status and legal climate. | 7.5 | NA |
| N. Pattison, S. M. Carr, C. Turnock and S. Dolan  2013 | 'Viewing in slow motion': patients', families', nurses' and doctors' perspectives on end-of-life care in critical care | While several studies have qualitatively explored the critical illness experience related to end of life (EoL), these tend to focus on others’ experiences, notably nurses. Given understandable methodological impossibilities of studying dying critically ill patients, nearly all of whom will be unconscious or sedated, none have been published that include in-depth experiences of dying critically ill patients. The studies described in the literature emphasise the lack of a complete picture of end-of-life care in critical care, and this study sought to explore this picture from each experience and perspective. | 1. Dual prognostication – the impact of a cancer diagnosis and an inevitably impending death 2. Meaning of decision-making – Participants and difficulties of making decisions 3. End-of-life care practices: choreographing a good death – the practices of doctors that result in a ‘good death’ as perceived by doctors and families | An important and original aspect of this research was the dilemma of treating patients over families at EoL in critical care, which was particularly evident in the data from all of the groups. This dilemma was particularly evident when deciding to withdraw and how to time and control the dying process.  The final theme indicated how there were opportunities for nurses to be pro-active in EoLC, but these were not always seized for a number of reasons, including a lack of confidence in cancer care and ethical knowledge. | NA | 18 |
| J. G. R. Ramos et. al  2019 | Withholding and Withdrawal of Treatments: Differences in Perceptions between Intensivists, Oncologists, and Prosecutors in Brazil | Legal concerns have been implicated in the occurrence of variability in decisions of limitations of medical treatment (LOMT) before death.  In this study, we aimed to survey a group of physicians (intensivists and oncologists) and prosecutors to assess differences in perception toward withholding and withdrawal of treatment. Specifically, we tried to evaluate perceived differences between withholding of active disease treatment (chemotherapy) and withholding of life-sustaining treatment (mechanical ventilation, MV). We also tried to assess per- ceived differences between withholding and withdrawal of life-sustaining treatment. | 1. Case 2 had the highest degree of agreement among intensivists: Withhold of mechanical ventilation (MV) with consent 2. A majority of intensivists agreed that Case 4 has grounds for criminal investigation 3. Case 4 was found to be the hardest case to answer among intensivists 4. Majority of intensivists agreed with withholding of active cancer treatment (Case 1) and with withholding of MV (Case 2) 5. There is a statistical difference in intensivists who agreed with withholding of MV and withdrawal of MV   *Summaries only relevant to ICU doctors |  | 6.5 | NA |
| A. Robertsen, E. Helseth, J. H. Laake and R. Førde, 2019 | Neurocritical care physicians' doubt about whether to withdraw life-sustaining treatment the first days after devastating brain injury: an interview study | BACKGROUND: Multilevel uncertainty exists in the treatment of devastating brain injury and variation in end-of-life decision-making is a concern. Cognitive and emotional doubt linked to making challenging decisions have not received much attention. The aim of this study was to explore physicians´ doubt related to decisions to withhold or withdraw life-sustaining treatment within the first 72 h after devastating brain injury and to identify the strategies used to address doubt. | All physicians described feelings of doubt.  Common strategies applied by physicians across specialities when dealing with uncertainty and doubt were:   1. Provision of treatment trials 2. Using time as a coping strategy- Distinguish cases that the right thing is to withdraw 3. Collegial counselling and interdisciplinary consensus seeking 4. Framing decisions as purely medical- The wishes and will of the patient was absent from their early decision-making   Variability between individual physicians   1. A few stated that they should not let their doubt dominate. They focused on their professional responsibility and how to apply their best possible situational judgement purely built on available information and overall medical judgement. 2. Others felt very humble and afraid to err because they had experienced how their best professional judgement had been wrong. 3. Some described how their own feelings, especially in cases with small children, influenced their judgement and how they sometimes identified with patients or families to a degree that threatened their objectivity. 4. Treatment intensity was perceived as being in part tied to their personality and temperament and in part to their level of experience 5. They also described how they sometimes felt at risk of being biased by cases they themselves had been exposed to, particularly cases with unexpected outcomes. | Institutional culture, ethics climate and individual physicians´ values, experiences and emotions seemed to impact judgements and decisions.  The collegial discussions helped physicians cope with stressful/negative emotions in circumstances of uncertainty.  Another issue is the conscious or subconscious risk of amplification of shared values as a result of group dynamics within a hospital subculture. Consensus in an interdisciplinary team is no guarantee of good decision making  A good ethics climate is built on a culture of ethical awareness and open interdisciplinary reflection. Physicians need to be self-reflective about their role as decision-makers. Acceptance of doubt is, as we see it, an essential part of a good ethics climate and is necessary for high-quality care.  Emotions and attitudes should be shared openly. Different opinions and values towards end-of-life issues must be expected and should be tolerated. Physicians in charge should help team members settle their differences  A process of reasoned discussions, elucidation of facts and exploration of values are worthwhile even if agreement is not forthcoming. Not one, but a range of reasonable decisions may be acceptable. Professionals should agree to respect views that they do not personally share.  Share and validate emotional dimensions, and allow them to be viewed as resources that shield light on end-of-life decisions | NA | 12 |
| R. E. Schutz et. al  2017 | Is There Hope? Is She There? How Families and Clinicians Experience Severe Acute Brain Injury | While all severe illnesses can affect personhood, the nature of neurologic illness, particularly in the ICU setting, is profoundly threatening to our concept of ourselves and may therefore represent a particular challenge to ICU palliative care.  The goal of our study was to describe palliative and supportive care needs of patients and family members in the neuro-ICU from the perspectives of families, nurses, and physicians. | Two themes have emerged:   1. Support for hope – Hope is presented as an object that was either present or absent, and as an action used for support and coping.   Hope, as an object, is seen by some as a commitment to preserve hope, even if false or misguided, due to a concern that removing hope would disrupt families’ ability to cope.  Hope as an action, can be give or taken away in the form of prognosticating.   1. Preservation of personhood – 1. Loss of personhood; Patients’ personhood was threatened by their SABI, and the resulting lack of meaningful communication hindered physicians’ ability to develop meaningful relationships with them.   2. Preservation of patient’s personhood; Families appreciated when clinicians treated the patient as a person, interpreting their actions as compassionate and caring. Conversely, families criticized clinicians who disregarded or devalued the patient  3. Personal support of families - Physicians recognized the importance of providing emotional support, but felt restrained by a lack of time and patient load. | The quotes presented in this article highlight the continued need for serious illness communication training for clinicians and suggest distinct challenges in the care of patients with severe acute brain injury (SABI) and their families.  In this study, uncertainty prompted families to rely on hoping to support their coping ability and resilience, but left physicians unsure whether to support or question hope.  Families wished for clinicians to acknowledge and treat both patients and themselves as persons, which was achieved through actions such as acknowledging the comatose patient, providing basic human comforts, and including families as part of the team. | NA | 15 |
| A. Simmonds  1996 | Decision-making by default: experiences of physicians and nurses with dying patients in intensive care | Modern medical treatments, coupled with society's denial of death, make it difficult to die in an urban teaching hospital. An increasing number of difficult ethical dilemmas and choices centre around the type and number of interventions to offer to critically ill patients.  The purpose of this study was to explore the impact on ICU physicians and nurses of caring for dying patients whom they perceive to be over or undertreated. This knowledge was sought in an effort to make care for the dying more humane for all those involved. | 1. Related to death:    1. Difficult to talk and accept death    2. Feeling of failure at the death of their patients    3. Daily routine of ICU allows them to avoid or ignore death    4. Feeling of personal failure fades with experience and maturity 2. Related to overtreatment    1. Feeling of powerlessness    2. Does not want aggressive care for themselves or their loved ones when it is time to die 3. Reasons for overtreatment    1. Fear of litigation    2. Unrealistic expectation from patient’s families    3. Unclear healthcare providers do not give realistic expectations    4. Caught up with technology    5. A natural death allows them to avoid making EoL decision    6. Uncertainty of when treatment is futile | Failure to facilitate or make prompt decisions regarding end-of-life care that will allow patients and families to choose a peaceful death represents "decision-making by default."  For some, deciding to discontinue aggressive therapy and life support feels like "playing God." They believe they are deciding to end the patient's life and it is not easy to live with the conviction that you have failed or have "caused" a person's death. For a physician, who experiences the death of a patient as a personal loss or "hurt," death becomes an enemy to be resisted with all possible means. Also, when one has the decision-making responsibility, it is difficult to make the judgment that further active treatment is futile. | NA | 10 |
| M. Svantesson, P. Sjökvist and H. Thorsén  2003 | End-of-life decisions in Swedish ICUs: how do physicians from the admitting department reason? | A major problem in ICU care is to define the turning point, when meaningful treatment becomes futile  This study’s purpose was to explore how admitting-department physicians reason when they make end-of-life decisions for ICU patients. Consequently, we studied a continuous series of clinical decisions rather than specifically asking about ethically difficult situations. | When describing their approach to decision making processes, these main points were seen:   1. 2 major approaches were expressed – 1. Explore curative treatment until realising patient will die despite life support 2. Save life while actively considering limiting life support 2. Differences in how active physicians were in decision-making   This study also identified a pattern in the decision making process:   1. Defining their role and contact with the patient – Primary or consultative responsibility 2. Knowledge of the patient – Patients’ present and former physical status were especially important 3. Evaluation and action – Uncertainty in prognosticating and deciding on intent of management 4. Turning point – mostly unanimous discussions with other physicians 5. Decision-making – ownership of decision | Personal characteristics of the physician seems decisive for power in decision-making  Physicians did not mention discussions with nurses. This is similar to most findings in earlier studies, which show low involvement of nurses in end-of-life issues.  EoL decisions, which are expected to be based on ethical considerations, were in reality made on medical grounds and were made by physicians.  a stricter organisation of interdisciplinary conferences would enable nurses to participate in decisions. The nurse’s knowledge of the patient (continuity of care granted) may complement a medical decision, which may because of this, become an ethical decision for a patient who is incompetent. | NA | 16 |
| C. Teixeira, O. Ribeiro, A. M. Fonseca and A. S. Carvalho  2014 | Ethical decision making in intensive care units: a burnout risk factor? Results from a multicentre study conducted with physicians and nurses | Working in IC has been shown to have high levels of burnout.  Burnout can be understood in terms of an ethical framework: on the one hand, burnout has an impact on the professionals themselves, and this may increase their vulnerability; on the other hand, considering depersonalisation, burnout may increase patients’ and relatives’ vulnerability. Another aspect for consideration refers to the ethical responsibility for preventing burnout in healthcare professionals.  Therefore, it seems relevant to study the ethical problems that may influence burnout syndrome development in physicians and nurses working in IC settings. | Burnout subdimensions defined were; Emotional exhaustion (EE), depersonalisation (DEP), and lack of personal and professional achievement (PPA).  Relevant associations were found between burnout sub-dimensions and job responsibilities:   1. Physician, in general, more frequently make ethical decisions in IC 2. A positive association was not found between burnout and need to withdraw treatments, to withhold treatments or to proceed to terminal sedation 3. No association between ethical decisions and burnout subdimensions 4. No association between need to make ethical decisions and DEP   *Summaries only relevant to ICU doctors | Withholding and withdrawing treatment may be seen as passive and active actions respectively, resulting in a greater decision-making burden in the former as withdrawing tend to involve others.  The need to make ethical decisions, and the way in which the ethical decisions were taken, may play a major role in the increase in burnout.  When ethical issues were presented, if the process of decision was taken without necessary ethical deliberation and without the right involvement of all the team, this could have a negative impact among the professionals.  It is fundamental to clarify professionals’ roles and practices, that they are supported in those roles and are responsible for continuing to provide acceptable care at the end of life. Workplace strategies for dealing with ethical dilemmas could be an important factor in reducing burnout.  In an era marked by increasing concern regarding the scarcity of ICU resources, the ethical issues and tensions analysis are of paramount importance in providing quality of care to patients and in reducing staff turnover. | 11.5 | NA |
| M. O. Tironi et. al  2016 | Prevalence of burnout syndrome in intensivist doctors in five Brazilian capitals | Burnout is related to the provision of services. Vulnerability to its development increases when this interaction involves a significant load of responsibility, protection and care for another, which occurs in intensivist work.  Our study aims to estimate the prevalence of burnout in intensivist doctors who work in adult, pediatric and neonatal intensive care units in five capitals representative of the Brazilian geographic regions with a substantial sample of these professionals. | The findings found:   1. The prevalence of burnout based on a high score in at least one dimension was 63.8% for doctors who worked in adult ICUs and 7.1% had high scores in the three dimensions simultaneously. 2. Of the three dimensions, high scores were observed in 7.1% of the doctors who worked in the adult ICUs   *Summaries only relevant to ICU doctors | The high prevalence of burnout may be due to work overload and an imbalance between technical and interpersonal preparation. We believe that there may be a gap in the psycho-emotional training of intensivists, such that the technical preparation, even when of high quality, may not be sufficient for the intensivists to deal with the day-to-day emotional demands of the ICU. This hypothesis is supported by the finding that three of the four factors considered most stressful concerned the relationship with patients and families. | 8 | NA |
| S. A. Trankle  2014 | Is a good death possible in Australian critical and acute settings?: Physician experiences with end-of-life care | The aim of this report is to document accounts of physicians in Australia who need to negotiate end-of-life care for patients and others in critical and acute settings. The broad-reaching interpersonal nature of managing death and dying is examined. Physicians’ experiences and their ability to assist “good deaths” are considered in relation to multiple contextualised influences. | Three key themes were identified:   1. Understanding a good death: Physicians’ perspectives – A good death is multifaceted and requires communication and involvement of patients, as well as collaborating physicians 2. Whose job is it to Palliate: A changing system of care – Unclear job expectations can cause job dissatisfaction and conflict among physicians 3. Negotiating appropriate care is a difficult experience – Conflict can occur with families, colleagues. Physicians can feel emotional pressure from families who negotiate for further intervention. | Patient and family care, and physician well-being and motivation, are all contingent on how specific and multitudinally influenced bedside dynamics can be negotiated.  Physicians often experienced helplessness, sadness and distress, particularly when being compelled to practice against their better judgement and without adequate skills. They further identified poor collegial collaboration and support, where a lack of professional awareness and understanding of cross-disciplinary expertise often compounded institutional impediments to care. | NA | 24 |
| R.-L. Van Keer, et. al  2019 | Challenges in delivering bad news in a multi-ethnic intensive care unit: An ethnographic study | Delivering bad news to patients and their families is challenging. Healthcare workers should be well prepared and communicate this in good time, repeatedly and carefully in an honest and clear manner. In this study, we aim to investigate ‘the delivery of bad news to patients and relatives from ethnic minority groups in the ICU’. Thereby we explore difficulties encountered by nurses and physicians before and during the actual communication of bad news and strategies used by them to deal with these difficulties. | The results identified physicians’ procedure for delivering bad news and its difficulties in choosing suitable conversation partner(s), choosing the place of conversation, and the information exchange itself.   1. Difficulties in choosing suitable conversation partner(s) – Choosing the appropriate family member or legal representative or a large group of relative or matching gender were strategies used by physicians 2. Choosing the place of conversation – Normalizing bedside conversations 3. Information exchange – physicians faced denial, lack of comprehension, aggression. 6 strategies physicians used were identified: 1. Multiple intensive conversations, adapted to the family situation   2. Seeking assistance of a colleague & actively supporting a colleague 3. Avoiding further communication with relatives 4. Giving hope to relatives 5. Ad hoc interpreter 6. Security agents | There is a risk of dealing with these problems in a quick and ad-hoc manner, while it is more appropriate to do this in a structured manner. This can have negative consequences for all parties involved (e.g. tensions between staff, endangering of care for patients and relatives).  A well-thought out protocol for the delivery of bad news in a multi-ethnic ICU is required, consisting of clear agreements on, e.g. the choice of conversation partners, information exchange and the documentation of the communication in patients’ medical files. Practical measures should be sufficiently discussed with patients from ethnic minority groups and their relatives. | NA | 16 |
| I. Wåhlin, A.-C. Ek and E. Idvall  2010 | Staff empowerment in intensive care: Nurses’ and physicians’ lived experiences | There are a few studies on workplace empowerment in intensive care, however these are based on questionnaires and more knowledge is needed about what intensive care staff consider empowering as told in their own words. Empowerment is reflected in this study as experiences of inner strength and power.  The aim of this study was to describe empowerment from the perspective of intensive care staff – What makes intensive care staff experience inner strength and power? | 3 general structures and 1 typological structure are described:  General structures:   1. Feelings of doing good – Inner satisfaction when good care was delivered and is frustrating when good care is not delivered. 2. Nourishing encounters – Getting to know a patient’s story contributed to shaping a physician’s personality 3. Teamwork – it was empowering when team members shared the same goals   Typological structure:   - A communicative work culture was seen to be important   *Summaries only relevant to ICU doctors | It was expressed as disempowering when delivered care was not experienced as good, such as when there was a lack of discussion and/or consensus about end-of-life decisions.  Attitudes regarding how long treatment should go on in such situations often differed between various professionals, with physicians frequently wanting to continue therapy longer than nurses and enrolled nurses wished. This may be because it is physicians who make the final decision, at the same time as they are confronted with the patient’s pain and anxiety to a lesser extent than nurses and enrolled nurses are. | NA | 13 |
| S. D. Walter et. al  1998 | Confidence in life-support decisions in the intensive care unit: a survey of healthcare workers. Canadian Critical Care Trials Group | We previously reported the results of a survey of Canadian healthcare workers that demonstrated extreme variability in decisions regarding withdrawal of life support in response to the same patient care scenarios.  This study aims to describe the relation between ICU healthcare worker choices about whether to withdraw life support, and their confidence in making those choices. | These findings were relevant to ICU doctors:   1. Intensivists considered themselves very confident in their decisions 40% of the time, moderately confident 47% and minimally confident 13% of the time 2. ICU intensivists were less aggressive in their choice of care than ICU nurses and ICU house staff 3. High confidence levels were often associated with extremes of level of care decisions.   *Summaries only relevant to ICU doctors | This cross-sectional survey has only established an association between level of care and confidence. A possible reason may be that clinicians are more confident with extreme decisions, although the confidence is unwarranted.  A second more plausible explanation may be that an intermediate level of care is chosen when clinicians are unsure of the correct course of action. A third alternative may be that healthcare workers report greater confidence when they take extreme measures, to reduce the cognitive dissonance between what they feel they must do and their actual uncertainty about the results of their actions. | 13 | NA |
| L. Weng et. al  2011 | Attitudes towards ethical problems in critical care medicine: the Chinese perspective | Although culturally Chinese, Hong Kong has a unique colonial history and the ethical attitudes and behaviour of ICU doctors in China may be quite different from those of Western or Hong Kong ICU doctors.  In a cross-cultural environment, failure to identify appropriate moral and ethical expectations may lead to impractical or unrealistic behavioural rules, interpersonal conflict or societal disapproval  This survey had two objectives: firstly, to document current attitudes and practices of ICU doctors in China when dealing with issues that have strong ethical and moral dimensions; and secondly, make comparisons with those reported by ICU doctors in Hong Kong and Europe. | A few key themes were highlighted from their multiple choice questionnaire:  Admission practices – limited by bed availability. However, patients with poor chances of survival would still be admitted  Information transfer and communication – majority would provide information based on patient’s condition, prognosis and level of education. Only 28% would disclose all details of an iatrogenic incident.  DNR orders – Most responses indicated DNR orders were not frequently applied. The majority believed that if used, it should be discussed with the patient or relatives.  Limitation of life-sustaining therapy (LOT) – Most responses indicated the practice of LOT was relatively uncommon. The majority of respondents indicated they were seldom or never at east when talking to family representatives about LOT and that they sometimes or often received requests from relatives for inappropriate support in a terminally ill patient.  Active shortening of the dying process (SDP) - In hopeless patients, 15% of respondents have sometimes, often or always ‘‘deliberately given large doses of drugs intentionally, until death ensues.  Legal aspects relating to end of life - Most respondents believed they would be exposed to personal risk when following DNR orders or LOT, but only 36% when declaring brain death.  The majority believed that legislation should determine the practice of all end-of-life issues. | The majority of doctors indicated they would provide specifically tailored information to patients, rather than complete medical information. This was also true for reporting iatrogenic incidents to patients or family. This response is in keeping with Chinese cultural approaches and previous observational data in Chinese doctors practising outside intensive care.  It is suggested that the importance of rescuing at any cost potentially creates conflict for the physician, who may believe that LOT is in the patient’s interests but fears that discussion with the family would create circumstances for disharmony, with harmony being a value that doctors are traditionally expected to uphold.  The fear of legal challenge may also contribute to Chinese doctors’ reluctance to deal with conflict relating to LOT at the end of life  Doctors themselves only reported making LOT decisions less than half the time, even though the majority believes that they should also make such decisions. This response contrasts with the commonly held view that traditional Chinese societies are strongly paternalistic and generally defer to doctors’ instruction. | 8.5 | NA |
| S. Workman, et. al  2003 | Intensive care nurses' and physicians' experiences with demands for treatment: some implications for clinical practice | To our knowledge, no study has attempted to determine why the provision of futile treatment can be such a difficult problem for medical personnel.  Therefore, we obtained first-person narrative accounts of futile treatment to better understand this experience from the perspective of medical staff. From the essential features described by participants in this study, we propose several approaches to manage difficult aspects of demands for treatment. | 3 main sources of concerns were raised:   1. Dying Patients – The suffering of dying patients as seen by pain behaviour and/or ongoing injury such as ischemic limb necrosis, was very distressing for physicians. 2. Distressed Family Members and Prognostic Uncertainty - The distress of the family members of dying patients was witnessed by both nurses and physicians and was a significant cause of distress for physicians. 3. A Breakdown in the Relationship With Family Members – Loss of trust and a difficult relationship with family members resulted in unpleasant tension. A physician asked themselves ‘Am I a monster?’ due to the families’ attitude towards them. | If trust already has begun to be lost, simple statements and questions such as, ‘I am concerned you don’t trust us. Why? What can we do to help?’ could be useful to start the process of relationship rebuilding. One physician suggested that finding a health care provider who is trusted also was useful.  As a first step to resolve distresses in situations of potential conflict, physicians should ask themselves 3 questions:  1. Has the importance of ensuring patient comfort and maintaining the patient’s bodily integrity been examined and discussed with family members?  2. Are family members extremely distressed? Do they require more emotional support? If so, has prognostic consensus been reached, or is the possibility of treatment withdrawal distressing because family members believe that survival remains possible?  3. Has there been a breakdown in the relationship between family members and medical personnel? What steps, besides providing treatment, can be taken to address this breakdown. | NA | 14 |
| S. C. Zambrano, A. Chur-Hansen and G. B. Crawford  2015 | On the emotional connection of medical specialists dealing with death and dying: a qualitative study of oncologists, surgeons, intensive care specialists and palliative medicine specialists | The frequency of death in palliative medicine can lead to depression and grief.  This paper reports on one particular aspect of the research findings, specifically: the experiences of medical specialists when developing an emotional connection with patients and how they cope with these experiences. | The ambivalence towards developing an emotional connection influenced participants’ experiences of the death of their patients.  The ambivalence was characterised by a dichotomy (one-half did not consider ICU doctors and was not included) and complemented by a third:   1. Getting emotionally involved - Allowing the expression of emotions and personal content resulted in a more effective relationship; treatments were deemed to be more effective and participants were transparent about their emotions.   Intensive care specialists wondered whether they might become more emotionally involved than they reported if patients were to spend more time in the intensive care unit.   1. Complementary: Finding a Balance –   Coping strategies were practiced regardless of medical specialty and included:  Being aware of their level of emotional engagement, protecting themselves, putting emotions into perspective, respecting the emotions of the patient’s family, overcoming their emotions without having time to grieve, and being busy  *Summaries only relevant to ICU doctors | Medical specialists who were unable to find a resolution to the conflict between emotional connection and detachment may face constant stress, potentially leading to burnout, compassion fatigue, mental health issues, risk behaviours and early retirement  More senior medical practitioners are frequently seen as role models. If senior clinicians are unprepared to reflect on their practice and share their emotional responses, junior medical specialists and those still in training may not learn these skills, thus perpetuating withdrawal from patients as an appropriate coping mechanism.  A possible intervention might be to encourage older generations of medical practitioners to discover the positive effects of recognising the emotional nature of their work, for example, through learning strategies such as reflective practice, self- awareness, self-control and situational awareness  Intensivists seemed to rely on their teams and find support in them. | NA | 20 |
