# Supplementary material for: The impact of caring for dying patients in intensive care units on a physician’s personhood: a systematic scoping review
Source: Philos Ethics Humanit Med. 2020 Nov 25;15:12. doi: 10.1186/s13010-020-00096-1 (PMC7685911; doi:10.1186/s13010-020-00096-1)
Supplement: Supplementary file 3 — Summary of Thematic Analysis and Direct Content Analysis with References. Themes and categories identified in analysis with references. (DOCX 714 kb) [file 13010_2020_96_MOESM3_ESM.docx]

*Additional file 3: Summary of Thematic Analysis and Direct Content Analysis with References*

| **Subthemes and examples** | **References** | **Categories and Example** | **References** |
| --- | --- | --- | --- |
| **Theme 1: Self** | | **Category 1: Innate Ring** | |
| **Emotion** | | **Perception of life and death** | |
| Internal conflict | (42, 49, 50, 67) | Confrontation with own mortality | (39, 60, 69, 70) |
| Management of own expectations | (66, 71, 72) | Conception of a good death impacting end-of-life care | (59) |
| Confrontation with own mortality | (39, 60, 69, 70) | One has a right to die | (36) |
| Apprehension/Distress |  | **Category 2: Individual Ring** | |
| - From end-of-life (EOL) care | (36, 39, 44, 46, 60, 61) | **Ability to make sense of things** | |
| - From communication with family | (41, 43) | Impact ability to make decisions | (44) (73) |
| - From belief that futile treatment prolonged dying process | (45) | **Abilities to communicate** | |
| - From possibility of litigation | (34, 64, 68, 70, 74-78) | Loss of ability to communicate and relate to patients | (33, 36, 37, 39, 42, 44, 52, 61, 69, 72, 78-80) |
| Fear due to unintentional transference to own family members | (40) | Improvement in communication skills | (37, 39, 40, 59, 61) |
| Satisfaction in providing end-of-life care | (40, 58, 59) | **Abilities to express feelings** | |
| **Thoughts** | | Emotional detachment | (36, 39, 44, 69, 72) |
| Doubt |  | Emotion connection | (40) |
| - Doubt in decision making | (44, 49, 63) | **Acquired ability** | |
| - Doubt in professional ability | (38, 49, 61, 63) | Lack of knowledge about end-of-life | (60, 65, 81) |
| - Doubtful in prognosis | (39, 45, 46, 49, 59, 73, 82) | Inadequate opportunities for end-of-life care training | (48, 65, 81) |
| - Doubt due to uncertainties in patient’s trajectories | (39, 42) | Doubt and lack of confidence in clinical skills | (39, 49, 65, 67) |
| Perception of emotional involvement |  | Testing of practical skills such as treatment withdrawal techniques | (83) |
| - As unhelpful | (44, 45, 59, 69) | Acquisition of new skills with experience | (39, 62, 63) |
| - As beneficial | (40, 69) | **Beliefs** | |
| Professional responsibility |  | *Personal Beliefs* |  |
| - To care for dying patients | (35, 37, 41, 44, 47, 49, 65-67, 73, 84) | Dilemmas about the balancing of opposing values | (49, 66, 67) |
| - To not cause death | (30, 37, 49, 59, 75, 85, 86) | Personal beliefs reflected in end-of-life practices and communication | (30, 35, 36, 49, 59) |
| - To care for patient’s family members | (44) | *Ethical dilemmas* |  |
| Death of a patient perceived to be a personal failure | (52, 60, 70, 86) | Differences in ethical opinion surrounding treatment withholding and withdrawal | (36) |
| Death of a patient not perceived to be a personal failure | (39, 60, 62, 70) | Futile treatment | (36, 60) |
| Perception of intervention |  | Lack of advanced directives and families’ aggressive care requests causing moral distress | (36, 42, 60) |
| - Prolongs suffering for patients | (34, 36, 45, 64, 66, 67, 79) | *Religious views* |  |
| - Prolongs suffering for patient’s family | (60) | Influenced end-of-life discussion and decision making | (80, 87) (81, 88) |
| - Withdrawal of treatment as life-shortening | (30) | Did not influence end-of-life practices | (31) (88) |
| Decision between active treatment or palliative intention | (39, 48, 66, 67) | **Perceived role as a doctor** | |
| Perception that nurses do not grasp the complexity of EOL decision making | (35, 66, 67) | Perceived duty to prolong life causing moral distress | (30, 37, 49) |
| Motivated to improve communication skills | (40, 59) | Uncertainty about role in end-of-life discussions resulting in no/late end-of-life discussion | (42, 66, 68) |
| Perception of intensive care unit as not conducive for palliative care discussions | (48, 59, 84, 89) | Paternalistic approach to decision making | (52, 60, 61, 90) |
| **Behavior** | | Satisfaction upon reconciling dual role of saving lives and managing death well | (59) |
| Impaired ability to make decisions | (37, 39, 40, 44, 59, 61, 73) | **Category 3: Relational Ring** | |
| Impaired ability to communicate | (33, 36, 37, 39, 42, 44, 52, 61, 69, 72, 78-80) | **Family** | |
| Emotional detachment | (38, 44, 45, 59, 60, 69, 70) | Fear due to unintentional transference to own family members | (40) |
| Difficulty and discomfort when broaching topic of death to patients | (33, 43, 61, 68, 80) | **Category 4: Societal Ring** | |
| Attempts to avoid discussion of death in general | (41, 79, 80, 88) | **Physical environment** | |
| Fear of litigation leading to defensive practice | (30, 34, 43, 64, 68, 70, 74-78, 90) | Availability of resources in different countries influencing end-of-life care | (31) |
| Adherence to decisions despite potential legal kickback | (34) | **Cultural environment** | |
| Factors affecting decision making |  | Physician’s end-of-life care attitudes, behaviors and decisions privy to cultural norms | (30, 33, 34, 43, 59, 75, 89) |
| - Experience | (35, 43, 63, 83) | Death and dying perceived as a “taboo” topic in certain cultures | (34, 41, 43, 68, 74, 80) |
| - Physician’s faith |  | Need for end-of-life care to be sensitive to different cultures encountered | (30, 33, 43, 80) |
| - - Influence | (60, 80, 87, 88) | Workplace culture impacting attitudes and practices | (32, 59, 85, 86) |
| - - Did not influence | (31, 88) | **Societal expectations** | |
| - Patient factors | (31, 50-52) | Societal expectations promoting survival and death prevention | (50, 59, 68, 86) |
| - Institutional factors | (33, 41, 48, 60, 65, 67, 81, 84) | Perception of treatment withdrawal as taking the life of one’s patient affecting physician’s end-of-life decision making | (30, 34, 35) |
| - Societal culture | (30, 33, 43, 59, 75, 89) | **Legal standards** | |
| Poor translation of spiritual ideas to goals of care | (88) | Fear of legal challenge affecting end-of-life care leading to defensive practice | (30, 34, 43, 64, 68, 70, 74-78, 90) |
| **Theme 2: Relationships** | | Adherence to decisions despite potential legal kickback | (34) |
| **Physician’s family** | | Unclear laws surrounding end-of-life practices breeding legal uncertainty | (34, 74, 78, 90) |
| Fear due to unintentional transference to own family members | (40) | **Professional Relationships** | |
| **Theme 3: Interactions** | | Conflict relating to end-of-life decisions with patient’s family and other healthcare professionals | (32, 35, 37, 44, 59-62, 65, 66, 68, 85, 86, 91) |
| **Patients** | | Positive professional relationships | (37, 49, 65) |
| Challenges during end-of-life communication | (41, 42, 44, 61, 66, 68, 74, 89, 90, 92) | **Professional standards** | |
| Managing expectations of patients | (49, 66, 71-73, 78, 79) | Professional expectation that doctors should not cause death or harm to patients | (30, 37, 49, 59, 85) |
| Inspiring interactions with patients | (40) | Responsibility of treatment withdrawal decision going against physician’s perceived professional standards | (37) |
| **Patient’s Family** | |  |  |
| Experiencing conflict with patient’s family | (32, 35, 37, 38, 41-43, 45, 46, 59-61, 65, 68, 70, 71, 74-76, 90) |  |  |
| Effects of conflict on the relationship | (42, 46, 91) |  |  |
| Family’s concern for patient's possible pain and distress | (30, 59, 60, 70, 75) |  |  |
| Managing expectations of patient’s family | (33, 37, 38, 45, 48, 60, 70) |  |  |
| Family’s distress after end-of-life care discussion | (61) |  |  |
| Empowering interactions with patient’s family | (40) |  |  |
| Factors affecting communication | (30, 32, 33, 41, 43, 44, 67, 71, 80) |  |  |
| Creation of soft landing when informing patient’s family about death | (30, 38, 40) |  |  |
| **Nurses & ICU Team** | |  |  |
| Conflict between physician and ICU nurses | (35, 37, 40, 44, 61, 62, 66) |  |  |
| Perception that nurses do not grasp the complexity of end-of-life decision making | (35, 66, 67) |  |  |
| Receiving support from other intensive care unit physicians in managing end-of-life decisions | (49) |  |  |
| **Physicians from other specialties** | |  |  |
| Challenges with interactions | (48, 49, 52, 59, 61, 66, 67, 69, 86) |  |  |
| Lack of understanding of one another’s role | (42, 66-68) |  |  |
| **Theme 4: Conflicts in providing end-of-life care** | |  |  |
| **Societal Culture** | |  |  |
| Societal culture impacting decision making | (30, 33, 43, 59, 75, 89) |  |  |
| Stigma associated with death or talking about death | (33, 41, 51, 92) |  |  |
| **Workplace Culture** | |  |  |
| Shapes the way doctors view death | (32, 49, 85, 86) |  |  |
| **ICU Environment** | |  |  |
| Suitability for palliative care teaching |  |  |  |
| - Not suitable | (65) |  |  |
| - Suitable | (81) |  |  |
| ICU as an inappropriate place to die |  |  |  |
| - Focus of care not allowing for palliative care | (59) |  |  |
| - Lack of privacy | (74) |  |  |
| **Legal environment** | |  |  |
| Uncertainty with regards to legal implications of end-of-life practice | (34, 90) |  |  |
| **Theme 5: Coping strategies** | |  |  |
| **Personal strategies** | |  |  |
| Effective communication to strengthen decision making position | (45, 49) |  |  |
| Confidence |  |  |  |
| - Gaining confidence through experience | (39, 40, 61) |  |  |
| - Gaining confidence with end-of-life discussion | (40, 62) |  |  |
| Taking breaks from ICU or practicing on other sites | (35) |  |  |
| **Strategies with patients** | |  |  |
| Collaboration with patients to reduce moral burden of decision making | (37, 68) |  |  |
| **Strategies with patient’s family** | |  |  |
| Creation of soft landing when informing patient’s family about death | (30, 38, 40) |  |  |
| Collaboration with patient’s family to reduce moral burden of decision making | (37) |  |  |
| **Strategies with colleagues** | |  |  |
| Conflict management interventions | (33, 37, 49, 64, 66) |  |  |
| Emotional and experiential sharing of caring for dying patients | (49, 65, 67) |  |  |
| Collaboration with interdisciplinary team members | (33, 37, 49, 66) |  |  |

**Additional References not found in Manuscript Main Text:**

70. Simmonds A. Decision-making by default: experiences of physicians and nurses with dying patients in intensive care. Humane health care international. 1996;12(4):168-72.

71. Amati R, Hannawa AF. Relational dialectics theory: Disentangling physician-perceived tensions of end-of-life communication. Health Commun. 2014;29(10):962-73.

72. Schutz RE, Coats HL, Engelberg RA, Curtis JR, Creutzfeldt CJ. Is There Hope? Is She There? How Families and Clinicians Experience Severe Acute Brain Injury. J Palliat Med. 2017;20(2):170-6.

73. Pattison N, Carr SM, Turnock C, Dolan S. 'Viewing in slow motion': patients', families', nurses' and doctors' perspectives on end-of-life care in critical care. Journal of Clinical Nursing (John Wiley & Sons, Inc). 2013;22(9-10):1442-54.

74. Almansour I, Seymour JE, Aubeeluck A. Staff perception of obstacles and facilitators when providing end of life care in critical care units of two teaching hospitals: A survey design. Intensive & critical care nursing. 2019;53:8-14.

75. Hawryluck LA, Harvey WR, Lemieux-Charles L, Singer PA. Consensus guidelines on analgesia and sedation in dying intensive care unit patients. BMC medical ethics. 2002;3:E3.

76. Asch DA, Hansen-Flaschen J, Lanken PN. Decisions to limit or continue life-sustaining treatment by critical care physicians in the United States: conflicts between physicians' practices and patients' wishes. Am J Respir Crit Care Med. 1995;151(2 Pt 1):288-92.

77. Ramos JGR, Vieira RD, Tourinho FC, Ismael A, Ribeiro DC, De Medeiro HJ, et al. Withholding and Withdrawal of Treatments: Differences in Perceptions between Intensivists, Oncologists, and Prosecutors in Brazil. Journal of Palliative Medicine. 2019;22(9):1099-105.

78. Fumis R, De Paula Pinto Schettino G, Domingos Corrêa T. Would you like to be admitted to the ICU? Intensivists' and the general public's preferences according to different outcomes. Intensive Care Medicine Experimental. 2018;6.

79. Barton E. Situating end-of-life decision making in a hybrid ethical frame. Communication & medicine. 2007;4(2):131-40.

80. Brooks LA, Bloomer MJ, Manias E. Culturally sensitive communication at the end-of-life in the intensive care unit: A systematic review. Australian Critical Care. 2019;32(6):516-23.

81. Chen E, McCann JJ, Lateef OB. Attitudes Toward and Experiences in End-of-life Care Education in the Intensive Care Unit: A Survey of Resident Physicians. The American journal of hospice & palliative care. 2015;32(7):738-44.

82. Cruz VM, Camalionte L, Caruso P. Factors associated with futile end-of-life intensive care in a cancer hospital. The American journal of hospice & palliative care. 2015;32(3):329-34.

83. Cottereau A, Robert R, le Gouge A, Adda M, Audibert J, Barbier F, et al. ICU physicians' and nurses' perceptions of terminal extubation and terminal weaning: a self-questionnaire study. Intensive Care Med. 2016;42(8):1248-57.

84. Tironi MO, Teles JM, Barros DS, Vieira DF, Silva Filho CM, Martins Junior DF, et al. Prevalence of burnout syndrome in intensivist doctors in five Brazilian capitals. Revista Brasileira de terapia intensiva. 2016;28(3):270-7.

85. Barnato AE, Tate JA, Rodriguez KL, Zickmund SL, Arnold RM. Norms of decision making in the ICU: a case study of two academic medical centers at the extremes of end-of-life treatment intensity. Intensive Care Med. 2012;38(11):1886-96.

86. Aslakson RA, Wyskiel R, Shaeffer D, Zyra M, Ahuja N, Nelson JE, et al. Surgical intensive care unit clinician estimates of the adequacy of communication regarding patient prognosis. Crit Care. 2010;14(6):R218.

87. Cardoso T, Fonseca T, Pereira S, Lencastre L. Life-sustaining treatment decisions in Portuguese intensive care units: a national survey of intensive care physicians. Crit Care. 2003;7(6):R167-75.

88. Ernecoff NC, Curlin FA, Buddadhumaruk P, White DB. Health Care Professionals' Responses to Religious or Spiritual Statements by Surrogate Decision Makers During Goals-of-Care Discussions. JAMA internal medicine. 2015;175(10):1662-9.

89. Van Keer RL, Deschepper R, Huyghens L, Bilsen J. Challenges in delivering bad news in a multi-ethnic intensive care unit: An ethnographic study. Patient Educ Couns. 2019;102(12):2199-207.

90. Cohen S, Sprung C, Sjokvist P, Lippert A, Ricou B, Baras M, et al. Communication of end-of-life decisions in European intensive care units. Intensive Care Med. 2005;31(9):1215-21.

91. Azoulay E, Timsit JF, Sprung CL, Soares M, Rusinova K, Lafabrie A, et al. Prevalence and factors of intensive care unit conflicts: the conflicus study. Am J Respir Crit Care Med. 2009;180(9):853-60.

92. Barton E, Aldridge M, Trimble T, Vidovic J. Structure and variation in end-of-life discussions in the Surgical Intensive Care Unit. Communication and Medicine. 2005;2(1):3-20.
