# Supplementary material for: The impact of caring for dying patients in intensive care units on a physician’s personhood: a systematic scoping review
Source: Philos Ethics Humanit Med. 2020 Nov 25;15:12. doi: 10.1186/s13010-020-00096-1 (PMC7685911; doi:10.1186/s13010-020-00096-1)
Supplement: Supplementary file 4 — Combined Themes with References. Combined themes after the funneling process with references. (DOCX 768 kb) [file 13010_2020_96_MOESM4_ESM.docx]

*Additional file 4: Combined Categories/ Themes with References*

| **Subcategory** | **Elaborations** | **References** |
| --- | --- | --- |
| **Category/Theme 1: Innate Ring** | | |
| 1. Perception of life and death | Confrontation with own mortality | (39, 60, 69, 70) |
|  | Conception of a good death impacting end-of-life care | (59) |
|  | Death of patient perceived to be a personal failure | (39, 60, 69, 70) |
|  | Death of patient not perceived to be a personal failure | (39, 60, 62, 70) |
|  | Conflict about prolonging life as it prolonged suffering | (32, 34, 36, 45, 49, 60, 64, 66, 67, 79, 85, 86) |
|  | One has a right to die | (36) |
| **Category/ Theme 2: Individual Ring** | | |
| 1. Ability to make sense of things | Impaired end-of-life decision making | |
|  | - Personal factors | (37, 39, 40, 44, 59, 61, 73) |
|  | - Patient factors | (31, 50-52) |
|  | - Institutional factors | (33, 41, 48, 60, 65, 67, 81, 84) |
|  | - Societal culture | (30, 33, 43, 59, 75, 89) |
|  | Doubt | |
|  | - Doubt in end-of-life decision making | (38, 42, 44, 49, 61, 63) |
|  | - Doubt about assessment of patient’s prognosis | (39, 42, 45, 46, 49, 59, 73, 82) |
|  | - Doubt due to uncertainties in patient’s trajectories | (39, 42) |
|  | Internal conflict when balancing care goals | (42, 49, 50, 67) |
|  | Internal conflict when managing own expectations | (66, 71, 72) |
|  | Dilemmas about active treatment versus palliative intention | (48, 66, 67) |
| 2. Ability to communicate and relate | Loss of ability to communicate and relate to patients | (33, 36, 37, 39, 42, 44, 52, 61, 69, 72, 78-80) |
|  | Poor communication skills | (78, 80) |
|  | Difficulty and discomfort when broaching topic of death to patients | (33, 43, 61, 68, 80) |
|  | Attempts to avoid discussion of death in general | (41, 79, 80, 88) |
|  | Improvement in communication skills | (37, 39, 40, 61) |
|  | Confidence in ability to navigate difficult conversations | (39, 61) |
|  | Motivated to further improve communication skills | (40, 59) |
| 3. Ability to express feelings | Emotional detachment | (36, 38, 44, 45, 59, 60, 69, 70) |
|  | Emotion perceived as hinderance to job | (44, 45, 59, 69) |
|  | Apprehension/Distress | |
|  | - From end-of-life care | (36, 39, 44, 46, 60, 61) |
|  | - From communication with family | (41, 43) |
|  | - From belief that futile treatment prolonged dying process | (45) |
|  | - From possibility of litigation | (34, 64, 68, 70, 74-78) |
|  | Fear due to unintentional transference to own family members | (40) |
|  | Emotional involvement being considered as valuable | (40, 69) |
|  | Satisfaction in involvement in patient’s end-of-life care | (40, 58, 59) |
| 4. Acquired ability | Lacking knowledge about end-of-life | (60, 65, 81) |
|  | Inadequate opportunities for end-of-life care training | (48, 65, 81) |
|  | Doubt and lack of confidence in clinical skills | (39, 49, 65, 67) |
|  | Testing of practical skills such as treatment withdrawal techniques | (83) |
|  | End-of-life decision making differed with years of experience | (35, 43, 63, 83) |
|  | Acquisition of new skills with experience | (39, 62, 63) |
|  | Adequate end-of-life care training | (67) |
| 5. Beliefs | Personal Beliefs | |
|  | - Conflicting beliefs resulting in distress | (50, 66, 67) |
|  | - Dilemmas about the balancing of opposing values | (49, 66, 67) |
|  | - Personal beliefs reflected in end-of-life practices and communication | (30, 35, 36, 49, 59) |
|  | Ethical dilemmas | |
|  | - Ethical differences surrounding withholding and withdrawing | (36) |
|  | - Futile treatment | (36, 60) |
|  | - Lack of advanced directives and families’ aggressive care requests causing moral distress | (36, 42, 60) |
|  | Religious views | |
|  | - Influenced end-of-life discussion and decision making | (80, 81, 87, 88) |
|  | - Religion did not influence end-of-life practices | (31, 88) |
| 6. Perceived role as a doctor | To care for dying patients | (35, 37, 41, 44, 47, 49, 65-67, 73, 84) |
|  | Perceived duty to prolong life causing moral distress | (30, 37, 49) |
|  | Death of patient perceived to be a professional failure | (52, 60, 70, 86) |
|  | Death of patient not perceived to be a professional failure | (39, 60, 62, 70) |
|  | Uncertainty about role in end-of-life discussions resulting in no/late end-of-life discussion | (42, 66, 68) |
|  | Paternalistic approach to decision making | (52, 60, 61, 90) |
|  | Perceived professional duty to collaborate and care for needs of patient’s family | (30, 44, 48, 61) |
|  | Professional satisfaction from caring for dying patients | (40, 58) |
|  | Satisfaction upon reconciling dual role of saving lives and managing death well | (59) |
|  | Emotions perceived as hinderance to role as doctor | (44, 45, 59, 69) |
| **Category/Theme 3: Relational Ring** | | |
| 1. Family | Fear due to unintentional transference to physician’s own family members | (40) |
| **Category/Theme 4: Societal Ring** | | |
| 1. Physical environment | Availability of resources in different countries influencing end-of-life care | (31) |
|  | Intensive care unit environment as not conducive for end-of-life discussions | (48) |
|  | ICU as an inappropriate place to die | |
|  | - Lack of privacy | (74) |
|  | - Focus of care not allowing for palliative care | (59) |
|  | Suitability for palliative care teaching | |
|  | - Not suitable | (65) |
|  | - Suitable | (81) |
| 2. Cultural norms | Physician’s end-of-life care attitudes, behaviors and decisions privy to cultural norms | (30, 33, 34, 43, 75Trankle, 2014 #112, 89) |
|  | Death and dying perceived as a “taboo” topic in certain cultures | (34, 41, 43, 68, 74, 80) |
|  | Need for end-of-life care to be sensitive to different cultures encountered | (30, 33, 43, 80) |
| 3. Workplace cultural norms | Influencing views on death, end-of-life care attitudes, behavior and decision making | (32, 59, 85, 86) |
| 4. Societal expectations | Societal expectations promoting survival and death prevention | (50, 59, 68, 86) |
|  | Perception of treatment withdrawal as taking the life of one’s patient affecting physician’s end-of-life decision making | (30, 34, 35) |
| 5. Legal standard | Fear of legal challenge affecting end-of-life care leading to defensive practice | (30, 34, 43, 64, 68, 70, 74-78, 90) |
|  | Unclear laws surrounding end-of-life practices breeding legal uncertainty | (34, 74, 78, 90) |
|  | Adherence to decisions despite perceived potential legal kickback | (34) |
| 6. Professional Relationships | Patients | |
|  | - Challenges faced during end-of-life communication | (41, 42, 44, 61, 66, 68, 74, 89, 90, 92) |
|  | - Managing expectations of patients | (49, 66, 71-73, 78, 79) |
|  | - Inspiring interactions with patients | (40) |
|  | Patient’s family | |
|  | - Conflict between physician and patient’s family | (32, 35, 37, 38, 41-43, 45, 46, 59-61, 65, 68, 70, 71, 74-76, 90) |
|  | - Effects of conflict on relationship | (42, 46, 91) |
|  | - Family members concerned for patient's possible pain and distress | (30, 59, 60, 70, 75) |
|  | - Managing expectations of patient’s family members | (33, 37, 38, 45, 48, 60, 70) |
|  | - Family’s distress after end-of-life care discussion | (61) |
|  | - Empowering interactions with patient’s family members | (40) |
|  | - Factors affecting communication with family members | (30, 32, 33, 41, 43, 44, 67, 71, 80) |
|  | - Creation of soft landing when informing family about death | (30, 38, 40) |
|  | - Perception of intensive care unit as not conducive for palliative care discussions | (48, 59, 84, 89) |
|  | Nurses & ICU Team | |
|  | - Support from other intensive care unit physicians to help manage end-of-life decisions | (49) |
|  | Physicians from other specialties | |
|  | - Challenges with interaction | (48, 49, 52, 59, 61, 66, 67, 69, 86) |
|  | - Lack of understanding of one another’s role | (42, 66-68) |
| 7. Professional Standards | Professional expectation for doctors to not cause death or harm to patients | (30, 37, 49, 59, 85) |
|  | Responsibility to decide on withdrawal of treatment went against physician’s perceived professional standards | (37) |
| **Category/Theme 5: Conflicts in providing end-of-life care** | | |
| 1. Interpretation of duty of the physician | Professional expectation that doctors should not cause death or harm to patients | (30, 37, 49, 59, 75, 85, 86) |
|  | Responsibility of treatment withdrawal decision going against physician’s perceived professional standards | (37, 75) |
|  | Physician’s end-of-life care attitudes, behaviors and decisions | (30, 35, 37, 41, 44, 47, 49, 59, 65-67, 73, 75, 84-86) |
|  | Need for end-of-life care to be sensitive to different cultured encountered | (30, 33, 43, 80) |
| 2. Behavior of the physician | Doubts in self, conflicts in decision making | (30, 33, 38, 39, 42-46, 49, 59, 61, 63, 73, 80, 82) |
|  | Emotional and psychological overlay | (34, 36, 39, 41, 43-46, 60, 61, 64, 68, 70, 74-78) |
|  | Internal conflict between beliefs and duty | (30, 37, 49, 59, 85) |
| 3. Behavior of others | Conflict with intensive care unit nurses | (35, 37, 44, 49, 59, 66, 85) |
|  | Challenges with interactions with other professionals | (37, 40, 48, 49, 52, 61, 62, 66) |
|  | Perception that nurses do not grasp the complexity of end-of-life decision making | (59, 69, 86) |
| 4. Professional Standards | Conflict between respect for cultural norms and general practice | (30, 33-35, 41, 43, 66-68, 74, 75, 80, 89) |
|  | Conflict between team members on how to interpret way to proceed in grey situations | (32, 35, 37, 40, 44, 48, 49, 52, 59, 61, 62, 66, 67, 85, 86) |
| **Category/Theme 6: Coping strategies** | | |
| 1. Personal strategies | Effective communication to strengthen decision making position | (45, 49) |
|  | Confidence | |
|  | - Gaining confidence through experience | (39, 40, 61) |
|  | - Gaining confidence with end-of-life discussions | (40, 62) |
|  | Taking breaks from the intensive care unit or practicing on other sites | (35) |
| 2. Strategies with patients | Collaboration with patient to reduce moral burden of decision making | (37, 68) |
| 3. Strategies with patient’s family | Creation of soft landing when informing patient’s family about death | (30, 38, 40) |
|  | Collaboration with patient’s family to reduce moral burden of decision making | (37) |
| 4. Strategies with colleagues | Conflict management interventions | (33, 37, 49, 64, 66) |
|  | Emotional and experiential sharing of caring for dying patients | (49, 65, 67) |
|  | Collaborations with interdisciplinary team members | (33, 37, 49, 66) |

**Additional References not found in Manuscript Main Text:**

70. Simmonds A. Decision-making by default: experiences of physicians and nurses with dying patients in intensive care. Humane health care international. 1996;12(4):168-72.

71. Amati R, Hannawa AF. Relational dialectics theory: Disentangling physician-perceived tensions of end-of-life communication. Health Commun. 2014;29(10):962-73.

72. Schutz RE, Coats HL, Engelberg RA, Curtis JR, Creutzfeldt CJ. Is There Hope? Is She There? How Families and Clinicians Experience Severe Acute Brain Injury. J Palliat Med. 2017;20(2):170-6.

73. Pattison N, Carr SM, Turnock C, Dolan S. 'Viewing in slow motion': patients', families', nurses' and doctors' perspectives on end-of-life care in critical care. Journal of Clinical Nursing (John Wiley & Sons, Inc). 2013;22(9-10):1442-54.

74. Almansour I, Seymour JE, Aubeeluck A. Staff perception of obstacles and facilitators when providing end of life care in critical care units of two teaching hospitals: A survey design. Intensive & critical care nursing. 2019;53:8-14.

75. Hawryluck LA, Harvey WR, Lemieux-Charles L, Singer PA. Consensus guidelines on analgesia and sedation in dying intensive care unit patients. BMC medical ethics. 2002;3:E3.

76. Asch DA, Hansen-Flaschen J, Lanken PN. Decisions to limit or continue life-sustaining treatment by critical care physicians in the United States: conflicts between physicians' practices and patients' wishes. Am J Respir Crit Care Med. 1995;151(2 Pt 1):288-92.

77. Ramos JGR, Vieira RD, Tourinho FC, Ismael A, Ribeiro DC, De Medeiro HJ, et al. Withholding and Withdrawal of Treatments: Differences in Perceptions between Intensivists, Oncologists, and Prosecutors in Brazil. Journal of Palliative Medicine. 2019;22(9):1099-105.

78. Fumis R, De Paula Pinto Schettino G, Domingos Corrêa T. Would you like to be admitted to the ICU? Intensivists' and the general public's preferences according to different outcomes. Intensive Care Medicine Experimental. 2018;6.

79. Barton E. Situating end-of-life decision making in a hybrid ethical frame. Communication & medicine. 2007;4(2):131-40.

80. Brooks LA, Bloomer MJ, Manias E. Culturally sensitive communication at the end-of-life in the intensive care unit: A systematic review. Australian Critical Care. 2019;32(6):516-23.

81. Chen E, McCann JJ, Lateef OB. Attitudes Toward and Experiences in End-of-life Care Education in the Intensive Care Unit: A Survey of Resident Physicians. The American journal of hospice & palliative care. 2015;32(7):738-44.

82. Cruz VM, Camalionte L, Caruso P. Factors associated with futile end-of-life intensive care in a cancer hospital. The American journal of hospice & palliative care. 2015;32(3):329-34.

83. Cottereau A, Robert R, le Gouge A, Adda M, Audibert J, Barbier F, et al. ICU physicians' and nurses' perceptions of terminal extubation and terminal weaning: a self-questionnaire study. Intensive Care Med. 2016;42(8):1248-57.

84. Tironi MO, Teles JM, Barros DS, Vieira DF, Silva Filho CM, Martins Junior DF, et al. Prevalence of burnout syndrome in intensivist doctors in five Brazilian capitals. Revista Brasileira de terapia intensiva. 2016;28(3):270-7.

85. Barnato AE, Tate JA, Rodriguez KL, Zickmund SL, Arnold RM. Norms of decision making in the ICU: a case study of two academic medical centers at the extremes of end-of-life treatment intensity. Intensive Care Med. 2012;38(11):1886-96.

86. Aslakson RA, Wyskiel R, Shaeffer D, Zyra M, Ahuja N, Nelson JE, et al. Surgical intensive care unit clinician estimates of the adequacy of communication regarding patient prognosis. Crit Care. 2010;14(6):R218.

87. Cardoso T, Fonseca T, Pereira S, Lencastre L. Life-sustaining treatment decisions in Portuguese intensive care units: a national survey of intensive care physicians. Crit Care. 2003;7(6):R167-75.

88. Ernecoff NC, Curlin FA, Buddadhumaruk P, White DB. Health Care Professionals' Responses to Religious or Spiritual Statements by Surrogate Decision Makers During Goals-of-Care Discussions. JAMA internal medicine. 2015;175(10):1662-9.

89. Van Keer RL, Deschepper R, Huyghens L, Bilsen J. Challenges in delivering bad news in a multi-ethnic intensive care unit: An ethnographic study. Patient Educ Couns. 2019;102(12):2199-207.

90. Cohen S, Sprung C, Sjokvist P, Lippert A, Ricou B, Baras M, et al. Communication of end-of-life decisions in European intensive care units. Intensive Care Med. 2005;31(9):1215-21.

91. Azoulay E, Timsit JF, Sprung CL, Soares M, Rusinova K, Lafabrie A, et al. Prevalence and factors of intensive care unit conflicts: the conflicus study. Am J Respir Crit Care Med. 2009;180(9):853-60.

92. Barton E, Aldridge M, Trimble T, Vidovic J. Structure and variation in end-of-life discussions in the Surgical Intensive Care Unit. Communication and Medicine. 2005;2(1):3-20.
